# Design of a Selective Substrate and Activity Based Probe for Human Neutrophil Serine Protease 4

**DOI:** 10.1371/journal.pone.0132818

**Published:** 2015-07-14

**Authors:** Paulina Kasperkiewicz, Marcin Poreba, Scott J. Snipas, S. Jack Lin, Daniel Kirchhofer, Guy S. Salvesen, Marcin Drag

**Affiliations:** 1 Division of Bioorganic Chemistry, Faculty of Chemistry, Wroclaw University of Technology, Wyb. Wyspianskiego 27, Wroclaw 50–370, Poland; 2 Program in Cell Death and Survival Networks, Sanford-Burnham Medical Research Institute, 10901 North Torrey Pines Road, La Jolla, CA 92024, United States of America; 3 Dept. of Early Discovery Biochemistry, Genentech Inc., 1 DNA Way, South San Francisco, CA 94080, United States of America; Russian Academy of Sciences, Institute for Biological Instrumentation, RUSSIAN FEDERATION

## Abstract

Human neutrophil serine protease 4 (NSP4), also known as PRSS57, is a recently discovered fourth member of the neutrophil serine proteases family. Although its biological function is not precisely defined, it is suggested to regulate neutrophil response and innate immune reactions. To create optimal substrates and visualization probes for NSP4 that distinguish it from other NSPs we have employed a Hybrid Combinatorial Substrate Library approach that utilizes natural and unnatural amino acids to explore protease subsite preferences. Library results were validated by synthesizing individual substrates, leading to the identification of an optimal substrate peptide. This substrate was converted to a covalent diphenyl phosphonate probe with an embedded biotin tag. This probe demonstrated high inhibitory activity and stringent specificity and may be suitable for visualizing NSP4 in the background of other NSPs.

## Introduction

Neutrophils participate in the front line defense against microbial pathogens [1]. Their weapons include four active serine proteases: neutrophil elastase (NE), proteinase 3 (PR3), cathepsin G (CatG) [2] and the recently discovered proteinase 4 (NSP4, PRSS57) [3]. NSP4 along with the other three neutrophil serine proteases (NSPs) is stored in the azurophilic granules, but it is by far the least abundant NSP [3]. Although NSP4 is the newest member to be discovered, its evolutionary origin predates the other three NSP members, suggesting that NSP4 likely plays critical roles in neutrophil function. Furthermore, while other NSPs preferentially cleave after small aliphatic residues (NE and PR3) or large hydrophobic residues (CatG), NSP4 is distinct from the three other NSPs in its strict requirement for P1-Arg [3, 4]. Structurally, NSP4 presented a conundrum in the classical trypsin-chymotrypsin-elastase paradigm because the primary sequence surrounding the active site predicted an elastase-like specificity, yet it has trypsin-like specificity [3]. This conundrum was explained when the 3D crystal structure of the protease was solved, revealing that NSP4 has an occluded pocket and that the substrate P1 Arg side-chain adopts a noncanonical ‘‘up” conformation stabilized by a network of solvent-exposed H-bonds [4]. This configuration allows NSP4 to bind and efficiently hydrolyze substrates with Arg and Arg-derivative side-chain amino acids (citruline, methylarginine), but not those containing Lys [4]. The extended substrate specificity of NSP4 has been investigated by using a limited series of internally quenched synthetic substrates and a diverse peptide fragment library employing focused mass spectrometry [5, 6].

The different approaches to investigate proteases substrate specificity include positional scanning combinatorial peptide libraries [7, 8], mass spectrometry [9], and phage display methods [10]. However, these approaches are restricted to the diversity of 20 natural L-amino acids, which is often a challenge for engineering highly selective probes for discriminating proteases with similar substrate [11–13]. For endopeptidases such as NSPs, Hybrid Combinatorial Substrate Library (HyCoSuL) is the method of choice since it incorporates not just the diversity of natural L-amino acids, but also natural D-amino acids as well as a large number of unnatural derivatives, thereby exploring a much higher degree of conformational space than would be possible with natural amino acids. Previous HyCoSuL library scans yielded a highly active and selective substrates and activity based probe for the NSP NE [12], as well as specific substrates for human apoptotic caspases [13].

An activity based probe contains a visualization moiety (such as biotin) a specificity element (amino acid sequence) and an electrophilic warhead that covalently attaches to the catalytically active form of the enzyme [14, 15]. The main goal of this work was to develop a sensitive substrate and activity based probe for NSP4 with improved selectivity toward the other three neutrophil serine proteases–PR3, CatG, and NE. By employing HyCoSuL we discovered an optimal NSP4 substrate that we used as a template to design a highly selective activity based probe.

## Materials and Methods

### Library synthesis

**Fig 1:**
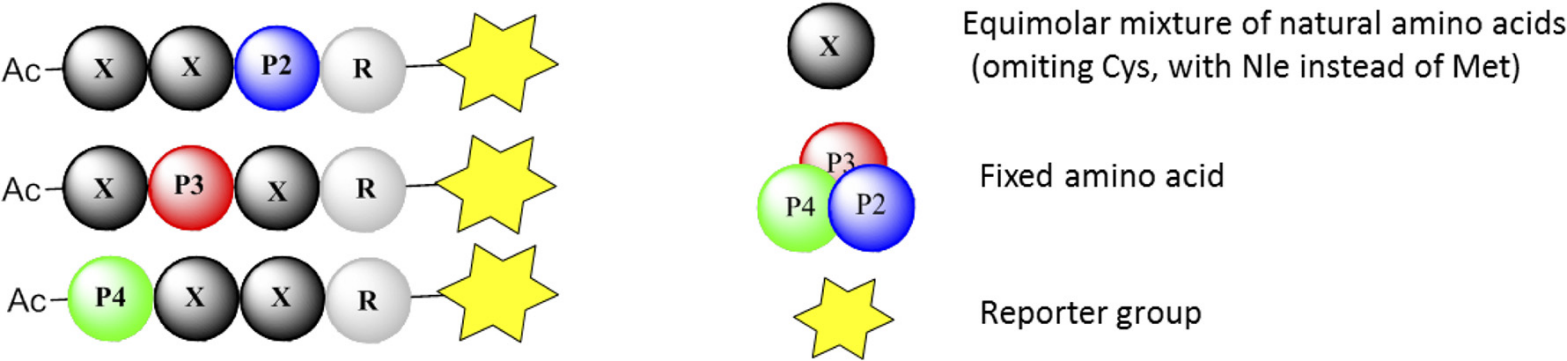
Scheme of the HyCoSuL P1 Arg library. The general library structure contains tetrapeptide derivatives with the sequence Ac-P4-X-X-Arg-ACC, Ac-X-P3-X-Arg-ACC, Ac-X-X-P2-Arg-ACC, where P4, P3 and P2 represents one of 120 fixed natural or unnatural amino acids and X represents an equimolar mixture of natural amino acids (omitting Cys and substituting Nle for Met) with ACC (7-amino-4-carbamoylmethylcoumarin) as a reporter group.

The Fmoc-protected acid form of the fluorogenic moiety ACC (7-amino-4-carbamoylmethylcoumarin) was synthesized according to Maly et. al. [16], and attached to Rink amide resin to allow solid phase synthesis [16]. The Fmoc-protecting group was then removed and Fmoc-Arg(Pbf)-OH (P1 position) was attached to the fluorogenic moiety. Because the yield of Fmoc-Arg(Pbf)-OH loading efficiency following double coupling is only about 50% [16], to protect remaining free amine groups, 3-nitro-1,2,4-triazole, AcOH and DICI in DMF were used, followed by DIPEA addition [16]. Then amine group of Fmoc-Arg(Pbf)-ACC-resin was deprotected, the resin was divided in 120 equal portions, and to each portion one fixed amino acid was added (Fig A in S1 Text). In the P3 and P4 positions an isokinetic mixture (X)–see Fig 1, containing natural amino acids (omitting Cys and substituting Nle for Met) was coupled [12, 13]. Fmoc-protecting groups were removed after each coupling reaction, the free N-terminal amine (at the P4 residue) was acetylated, followed by cleavage of the library from the resin. P3 and P4 sublibraries were synthesized in the same manner (for details see S1 Text). To clarify, Nle should properly be defined as an unnatural amino acid but was used in the natural amino acid isokinetic mixture to substitute for Met. It is included as an unnatural amino acid in the HyCoSuL (Fig 1).

### Substrate specificity determination–library screening

**Fig 2:**
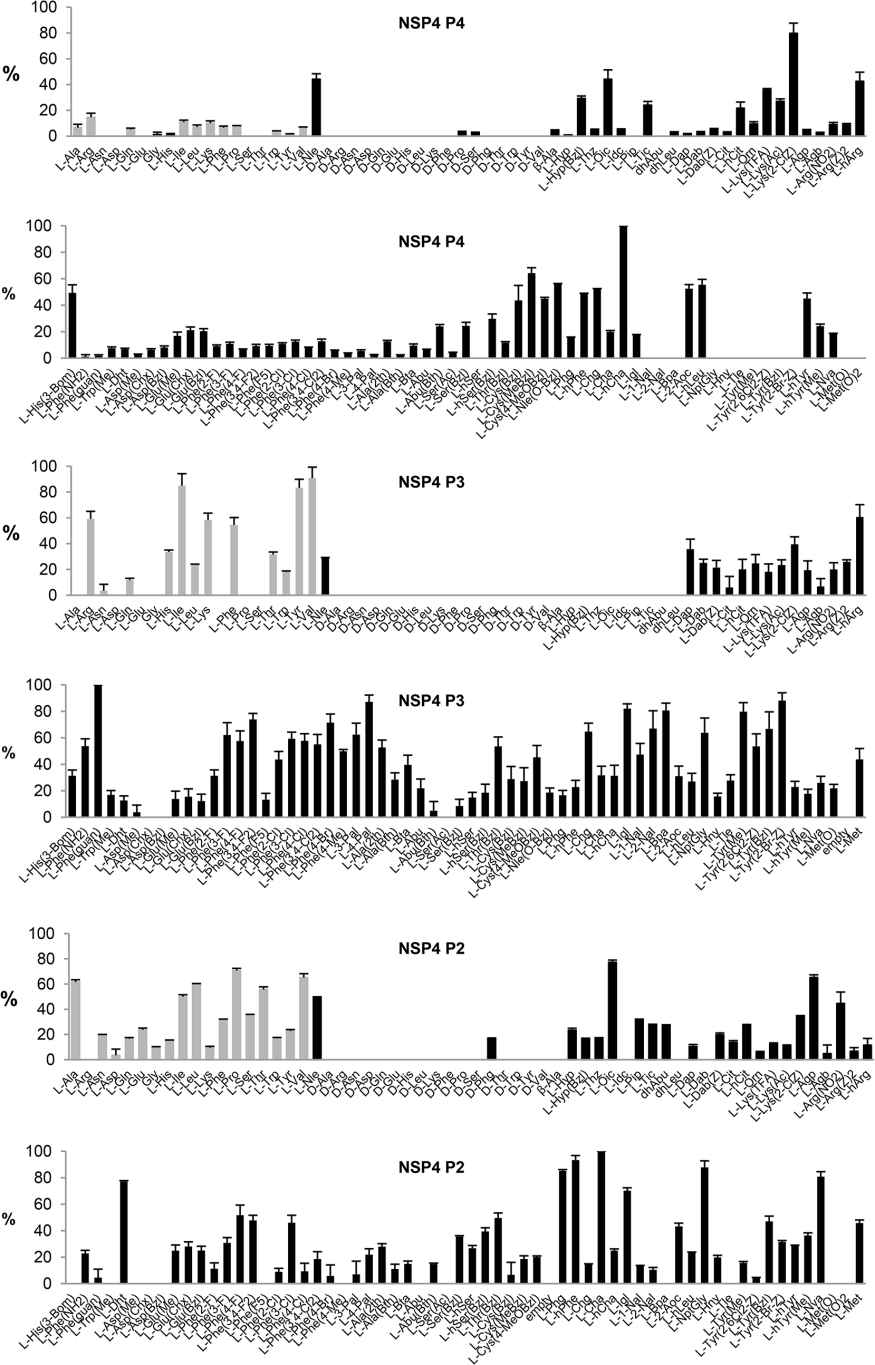
Determination of NSP4 substrate specificity. Preferences in the P4-P2 positions were determined by screening HyCoSuL, which contains tetramer peptides with the general structures Ac-P4-X-X-Arg-ACC, Ac-X-P3-X-Arg-ACC, Ac-X-X-P2-Arg-ACC, where P4, P3 and P2 represents fixed natural or unnatural amino acid and X represents an equimolar mixture of natural amino acids (omitting Cys and substituting Nle for Met). Screening was performed on a SpectraMax Gemini plate reader. Substrate hydrolysis rates were normalized to the most active component (100%) y axis. Natural amino acids are colored grey, unnatural black. Results are shown as an average of 3 experiments with S.D.

Each of the P2, P3, and P4 sublibraries was screened at 100 μM concentration with NSP4 in a final volume 100 μl. For the P3 sublibrary we employed 217.8 nM NSP4, and for the P2 and P4 sublibraries we employed 326.7 nM NSP4 to obtain robust signals. Initial rates of substrate hydrolysis were recorded as relative fluorescent units over time, and only the linear phase of substrate hydrolysis was used to determine the reaction velocity. From each library the highest value was normalized to 100% (Fig 2). For more details see SI.

### Optimal substrate and PK401 synthesis

#### Substrate

ACC was synthesized according to Maly et al. with small modifications [16]. Substrate synthesis was performed using a Fmoc-peptide synthesis method described above on Rink Amide Resin incorporating HATU/collidine and HOBt/DICI as a coupling reagents [16].

#### Activity Based Probe

**Fig 3:**
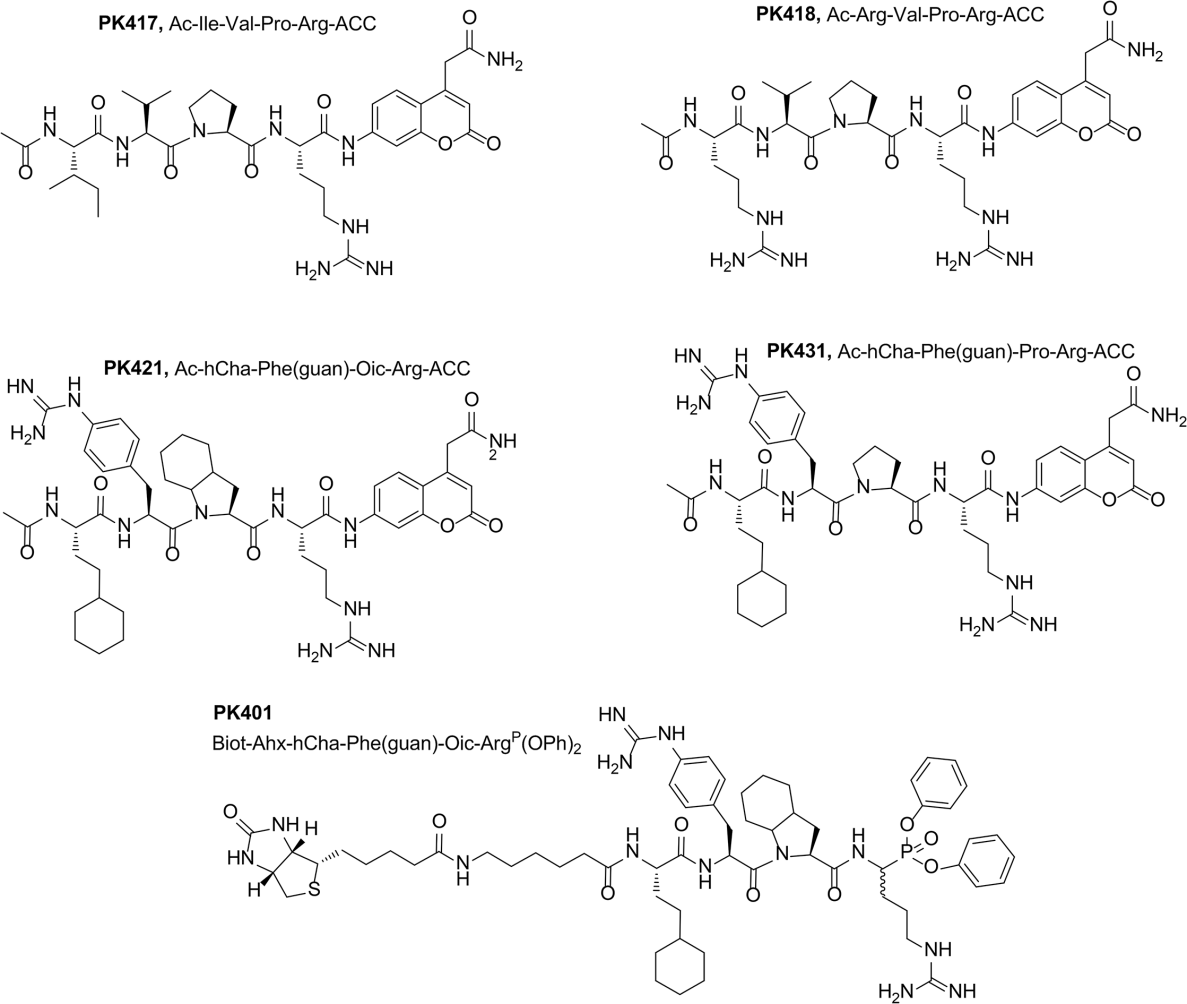
Structures of the optimized NSP4 substrates based on natural (PK417 and PK418) and natural/unnatural amino acids (PK421 and PK431). The activity-based probe (PK401), a diphenyl phosphonate derived from the optimal substrate sequence—PK421—is shown as the last structure.

We converted Ac-hCha-Phe(guan)-Oic-Arg-ACC into an activity based probe by replacing the ACC fluorophore with the electrophilic warhead diphenyl phosphonate and modifying the N-terminus of the peptide sequence with a biotin tag separated from recognition elements with 6-aminohexanoic acid [17–19]–see Fig 3. NH_2_-Arg(Boc)_2_^P^(OPh)_2_ was synthesized according to previous methodology [20]. Biot-Ahx-hCha-Phe(guan)-Oic-OH was synthesized in solid phase using 2-chlorotrityl chloride resin by performing a standard Fmoc-peptide synthesis procedure. Subsequently Biot-Ahx-hCha-Phe(guan)-Oic-OH and NH_2_-Arg(Boc)_2_^P^(OPh)_2_ were coupled in solution with HATU/collidine in DMF. Reaction completion was monitored by analytical HPLC and the crude product was purified on semi-preparative HPLC. Two main fractions–diastereoisomers were collected (PK401b and PK401). Finally, deprotection of guanidine-protected Boc groups was carried out in TFA/DCM. PK401 identity was confirmed by high-resolution mass spectrometry and purity by analytical MS (See S1 Text).

### Substrates kinetics

Kinetic parameters of selected substrates were determined in 50 mM Hepes, 0.1 M NaCl, 0.1%TRITON-X100, pH 7.45 assay buffer on 96 wells Corning plates. Measurements were performed for a minimum of 20 minutes. The excitation wavelength was 355 nm, and the emission wavelength was 460 nm, with a cutoff of 455 nm. Human NE and CatG were purchased from Biocentrum, Krakow, Poland and PR3 from Athens Research Technology, Athens, Georgia, USA. Recombinant human NSP4 was expressed and purified as previously described [4].

To 20μl of substrates dissolved in assay buffer at known concentration ranges (PK421 and PK431–320–1μM, PK417 and PK418–460–27μM), 80μl of NSP4 (2.9nM), preincubated for 20 minutes at 37°C in buffer, was added. K_m_, k_cat_ and k_cat_/K_m_ parameters/constants were calculated using GraphPad Prism and Excel software. Each experiment was repeated three times, and the results are presented as an average +/- S.D.

### Determination of inhibition parameters (k_obs_/I) of NSP4, CatG, NE and PR3 with PK401

The active concentration of each NSP was determined by PK401 titration in 50 mM Hepes, 0.1 M NaCl, 0.1%TRITON-X100, pH 7.45 assay buffer on 96 wells Corning plates. Rfu/s values were measured for minimum 20 minutes. The excitation wavelength was 355 nm, and the emission wavelength was 460 nm, with a cutoff of 455 nm. Under pseudo-first order conditions, a fixed amount of NSP4 (2.8 nM active enzyme), PR3, NE, or CatG (100 nM active enzyme) was mixed with PK401 at different concentration ranges starting from at least 2-fold excess over NSP4 (6.6–20nM) and 7-fold excess over NE, CatG and PR3 (0.7–50μM). Substrate concentration for NSP4 was above K_m_, and below K_m_ for CatG, NE and PR3 (Ac-Nle(O-Bzl)-Met(O)_2_-Oic-Abu-ACC for NE, Ac-Glu(O-Bzl)-Lys(Ac)-hPro(O-Bzl)-Abu-ACC for PR3, Suc-Ala-Ala-Pro-Phe-AMC for CatG and Ac-hCha-Phe(guan)-Pro-Arg-ACC for NSP4). Substrate and PK401 were mixed together at 37°C in assay buffer, and the reaction was started by enzyme addition (each separately) preincubated in the assay buffer at 37°C. Product formation (P) proceeded at an initial velocity (V) and was inhibited over time (t) at a rate of (*k*_*a*_): P = V/ka*(1-e^-k^a^*t^)+C, where *k*_*o*_ is defined as *k*_*obs(app)*_ *−*the apparent rate of inhibition in the presence of substrate. *k*_*obs*_ was determined separately for each probe concentration using non-linear regression analysis in GraphPadPrism software. The obtained values were plotted against probe concentration giving *k*_*obs(app)*_/I (apparent second order rate constant). k_obs_/I (absolute value of the second order rate constant) was then calculated from k_obs_/I = k_a_/I *(1+[S]/Km) [21].

### SDS-Page and Western Blot

100nM of NSP4 was incubated with competing inhibitor—Ac-IVPR-CMK for 30 minutes at 37°C and then treated with different concentrations of probe—PK401 (2000nM and 1000nM) for 20 minutes at 37°C. Separately, 100nM of NSP4 was incubated with five different concentrations of probe- 2000nM, 1000nM, 100nM, 10nM, and 1 nM for 20 minutes in 37°C. As a control 100nM PK401 was incubated with assay buffer.

Samples were denatured and run in 4–12% SDS PAGE following transfer to nitrocellulose membrane. The membrane was blocked with 1.5% BSA in TBST overnight at 4°C and treated with IRDye800CW Streptavidin (dilution 1:10000 with 1.5% BSA in TBST) for 45 minutes. Then enzyme was visualized using a Li-cor fluorescence imager and Odyssey Molecular Modeling Software.

The same methodology was used to visualize potential cross-reactivity of NSP’s with PK401. 100nM of each NSP was incubated with buffer or 100nM PK401, followed by SDS-page and transfer to nitrocellulose membrane. For more details see S1 Text.

## Results

In previous studies we utilized HyCoSuL to obtain highly selective substrates and probes for human NE [12]. Because of NSP4's strong preference for Arg in P1 [3, 4], we constructed a HyCoSuL library with this amino acid fixed at the P1 position. To investigate the NSP4 substrate specificity at the S4-S2 subsites, we incorporated 19 natural and 101 unnatural amino acids in each position of the libraries. Libraries were synthesized essentially as described previously [12, 13]. Unnatural amino acids were selected to cover a broad spectrum of possible interactions at each subsite—for example bulky structures (hPhe, Bip), proline derivatives (dhPro, Oic), acidic (hGlu), basic (Phe(guan)), aliphatic (Nva) or branched side-chains (Tle).

### Substrate specificity determination

#### P4 position

From a pool of natural amino acids, aliphatic residues (Leu, Ile) and Arg were preferred over the more bulky, hydrophobic Phe. The optimal unnatural amino acid at P4 was homocyclohexylalanine (hCha)—around 5 fold more active than the optimal natural amino acid (Arg). In addition, we observed that amino acids with elongated side-chains such as hArg, hCha, hPhe are three or more times more active than their shorter derivatives (Arg, Cha, Phe). These results suggest that the S4 subsite is able to recognize spacious amino acid residues. In addition, the S4 pocket is stereospecific since it did not recognize substrates with D-amino acids (Fig 2).

#### P3 position

P3 showed broader tolerance than P4, consistent with the fact that P3 residues are mostly solvent exposed on NSP4 and other trypsin-fold proteases in general [4]. The natural amino acids Val, Ile, Tyr, Arg, Lys and Phe were among the most active at the P3 position. The best unnatural amino acid was the phenylalanine derivative with a guanidine group in *para* position (Phe(guan)), however other phenylalanine derivatives were also quite well tolerated at the S3 subsite. Interestingly, Pro and its derivative did not seem to be tolerated at S3. Similar to S4, the S3 subsite is stereospecific and does not recognize D-amino acids (Fig 2).

#### P2 position

Among the natural amino acids there was a preference for Pro, Val, Ala and Leu, but other natural amino acids were also reasonably well tolerated, indicating that the S2 pocket has a broad substrate specificity. The preferred unnatural amino acids were Cha, hPhe, Phg (hydrophobic) and Oic, a Pro derivative. Interestingly, NSP4 tolerated D-Phg in the S2 pocket, although we detected no activity of other D-amino acids (Fig 2).

### Kinetic constants for NSP4 substrate determination

Based on the library screening results, we selected several of the most active amino acids in each position and synthesized several fluorogenic substrates (Fig 3). In addition, two substrates containing only optimal natural amino acids were synthesized (PK417, PK418). After substrate purification (HPLC) kinetic parameters (k_cat_, K_m_, k_cat_/K_m_) for the synthesized substrates were measured. Overall the library results predicted the best individual substrates, however we noted that the optimal amino acid did not always completely match the HyCoSuL prediction. For example, the predicted best amino acid at P2 was Cha, yet in individual substrates Oic was preferred to Cha at P2. Table 1 shows the kinetic constants for the two best substrates compared to the best natural amino acid containing substrates.

**Table 1:** Kinetic analysis of tetrapeptide substrates for NSP4. Results are shown as an average of a minimum of 2 separate experiments with S.D.

Code, sequence	K_m_ [μM]	k_cat_ [s^-1^]	k_cat_/K_m_ [M^-1^s^-1^]
**PK417**, Ac-Ile-Val-Pro-Arg-ACC	96 ± 16	0.2 ± 0.02	2100 ± 300
**PK418**, Ac-Arg-Val-Pro-Arg-ACC	67 ± 10	0.06 ± 0.003 x 10^−3^	960 ± 190
**PK421**, Ac-hCha-Phe(guan)-Oic-Arg-ACC	61 ± 9.1	2.0 ± 0.54	32000.0 ± 4500
**PK431**, Ac-hCha-Phe(guan)-Pro-Arg-ACC	53 ± 0.8	1.2 ± 0.017	23000 ± 11

Substrate analysis demonstrated that a small modification in substrate sequence from Pro in substrate PK431 to Oic in substrate PK421 increased the k_cat_/K_m_ value by 39%. The optimal Ac-hCha-Phe(guan)-Oic-Arg-ACC (PK421) substrate contained unnatural amino acids and is around 15 times more efficiently cleaved by NSP4 (k_cat_/K_m_ 32000 M^-1^s^-1^) then the best substrate based on only natural amino acids PK417 (k_cat_/K_m_ 2100 M^-1^s^-1^).

### Cross reactivity with other neutrophil serine proteases

In addition to NSP4, neutrophils contain three other structurally-related serine proteases NE, PR3 and CatG [1] that are also stored in azurophilic granules. To estimate the degree of eventual cross reactivity of the optimal substrate with these three serine proteases, we measured kinetic parameters for each enzyme on PK421. Table 2 demonstrates that Ac-hCha-Phe(guan)-Oic-Arg-ACC is highly specific for NSP4 and is only very weakly hydrolyzed by CatG. In the case of CatG we were unable to saturate the enzyme and therefore could not calculate the individual values of k_cat_ and K_m_. Lack of measurable activity toward PR3 and NE made this sequence an ideal candidate for the design of an activity based probe.

**Table 2:** Kinetic parameters/constants for the hydrolysis of Ac-hCha-Phe(guan)-Oic-Arg-ACC substrate by neutrophil serine proteases to three significant digits.

Neutrophil protease	K_m_ [uM]	k_cat_ [s^-1^]	k_cat_/K_m_ [M^-1^s^-1^]
NSP4	61 ± 9.1	2.0 ± 0.54	32000 ± 4500
CatG	-	-	477±0.9
PR3	NA	NA	NA
NE	NA	NA	NA

NA–no activity detected.

### Activity Based Probe Analysis and Validation

The inhibitory efficiency/potency of PK401 and PK401b toward NSP4 was tested by calculating k_obs(app)_/I (apparent second-order rate constants for inhibition) under pseudo first-order conditions. The substrate-independent values of k_obs_/I were calculated after taking into account K_m_ for the assay substrates (S) by adjusting for the factor 1+[S/K_m_] [21]. PK401 (k_obs_/I 3.8x10^6^ M^-1^s^-1^) demonstrated 40 times higher k_obs_/I than PK401b (k_obs_/I 0.093x10^6^ M^-1^s^-1^) revealing that PK401 is the optimal diastereoisomer (most probable with an R chiral carbon on Arg). Cross reactivity toward other NSPs was determined for PK401 by similar calculations of second-order rate constants. No inhibition of NE and PR3 by PK401 was observed, while inhibition of CatG by the PK401 was three orders of magnitude slower than NSP4 (Table 3), indicating high selectivity of PK401 toward NSP4.

**Table 3:** Inhibition rate constants of NSPs by Biot-Ahx-hCha-Phe(guan)-Oic-Arg^P^(OPh)_2_ (PK401).

Protease	Substrate	K_m_ [μM]	k_obs_/I [M^-1^s^-1^]
NSP4	Ac-hCha-Phe(guan)-Pro-Arg-ACC	53 ± 0.8	3.8 x10^6^
CatG	Suc-Ala-Ala-Pro-Phe-AMC	> 100 *	3.0 x 10^3^
PR3	Ac-Glu(OBzl)-Lys(Ac)-hPro(OBzl)-Abu-ACC	13.7 ± 0.67	NI
NE	Ac-Nle(OBzl)-Met(O)_2_-Oic-Abu-ACC	0.28 ± 0.08	NI

NI–no inhibition observed; K_m_ values relate to the substrate used for analysis,

* K_m_ for this substrate was above 100μM, the concentration used in the assay. AMC – 7-amino-4-methylcoumarin.

**Fig 4:**
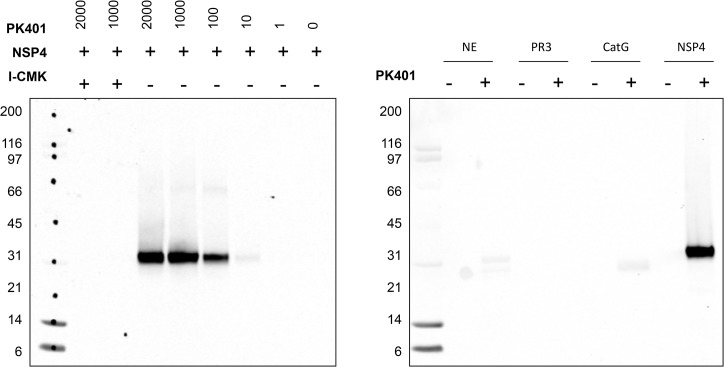
Visualization of PK401 with purified NSP4 and all NSP’s. (A) NSP4 was treated with PK401 in a range from 1 to 2000nM. (B) 100nM of NE, PR3, CatG and NSP4 with or without 100nM of PK401. (A, B) Samples were denatured in SDS sample buffer, run in SDS/PAGE followed by membrane transfer. The blot was developed with fluorescently-tagged streptavidin and imaged by fluorescence scanning (See S1 Text).

To analyze the sensitivity of the biotinylated activity based probe (PK401) we performed SDS-PAGE followed by nitrocellulose transfer and visualization with fluorescent streptavidin (Fig 4A). We were able to observe robust labeling of NSP4 at low probe concentration (10nM), 10 times less than enzyme concentration (100nM). Importantly, NSP4 active site blocking by a covalent chloromethyketone inhibitor (Ac-Ile-Val-Pro-Arg-CH_2_Cl) chemically-unrelated to PK401 prevented binding even at very high probe concentration (2000nM), revealing that the probe binds to the active site of NSP4, as predicted.

To further examine the selectivity of PK401 each NSP (100nM active enzyme) was incubated with PK401 (100nM) under identical conditions, and probe binding analyzed. Fig 4B demonstrates only trace amounts of labeling of NE and CatG and visible confirming the high selectivity of PK401 for NSP4.

## Discussion

Neutrophil serine proteases participate in the first line of host defense and are stored in neutrophil granules in an active form to be released during inflammatory responses. Three NSP’s: NE, PR3 and CatG have been known for decades [1, 2, 22]. In contrast, NSP4 has been more recently discovered and exists in neutrophil granules at a much lower abundance than its related NSPs [23]. NSPs can participate as beneficial enzymes in tissue remodeling, but are considered detrimental in the development of inflammatory diseases and possibly helping to seed the tumor microenvironment [22]. NE has been implicated in the formation of Neutrophil Extracellular Traps (NETs)–extruded nuclear material that is thought to ensnare pathogens [24]. NE decorates NETs, yet interestingly it does not appear to be active in this location and retains activity when cell-associated in granules, as evidenced by activity probe profiling [12, 25]. Currently, a role of NSP4 and other NSPs in NET formation remains unknown. The development of activity based probes specific for NSP4 will be helpful in determining NSP4 functions in NET formation and other neutrophil functions involving NSPs.

Previous approaches to define the substrate specificity of NSP4 have shed light on a limited number of properties, the most intriguing of which is its preference for Arg and Arg derivatives in P1 [3–6]. A previous report has also identified that NSP4 is able to recognize Phe, Ala, Ser, Asn, Glu, Arg and Gly residues in P4 [6], which is consistent with our results. Interestingly, we did not observe significant cleavage of substrates containing Asp or Gly at P4 as previously reported [5]. However, the lack of normalized quantitation with these previous reports makes it difficult to compare datasets.

HyCoSuL screening demonstrated that the S2 and S3 subsites tolerate a broad range of amino acids. The most stringent, specificity defining subsite apart from P1 appears to be P4, as demonstrated by the narrow range of amino acids tolerated. These data correlate with the previously described partial specificity profile for NSP4 obtained with a focused proteomics approach [3] and partially with a fluorescence quenched substrates library, where P4/P3 amino acid pairs in P4/P3: Phe/Lys, Ala/Asn and Ala/Lys was described as the best recognized [6]. Our report provides the first comprehensive substrate specificity profile of NSP4. It is intriguing that HyCoSuL predicted the best unnatural amino acid at P2 to be Cha, but our synthesis of individual substrates revealed that Oic at this position was preferred. This likely has two explanations. First, the Cha substrate may have some interactions between the hCha (P4) and Cha (P2) that would constrain the peptide from binding in an appropriate conformation–so called negative subsite cooperativity [26, 27]. Second, neutrophil serine proteases have a tendency to prefer Pro in P2 [22], which would explain the potency of the Pro derivative Oic in P2. This finding demonstrates the importance of investigating the lead hits of HyCoSuL by validating individual sequences. Importantly, HyCoSuL gives leads, but synthesis of individual substrates around these leads provides the best substrates. Thus, HyCoSuL is invaluable starting point leadings towards identification of optimal substrates.

Employing unnatural amino acids in substrate design led to an enhanced substrate kinetic efficiency (about 15 fold) compared with the best natural sequence, and coincidently eliminated undesirable cleavage by other NSPs. Several fluorescence quenched substrates based on natural amino acids have been described, but these substrates cannot be converted to activity based probes. Our approach enabled us to synthesize a highly active [3.8 x 10^6^ M^-1^s^-1^] NSP4 PK401 probe (Biot-Ahx-hCha-Phe(guan)-Oic-Arg^P^(OPh)_2_) which provides the possibility to image even small amounts of NSP4. PK401 is the first specific activity-based probe synthesized for NSP4 showing no activity toward NE and PR3 and only minimal activity toward CatG. It was important for us to examine cross-reactivity with CatG since this protease is known to demonstrate dual trypsin-like and chymotrypsin-like specificity at P1 [28] and, therefore, could react with probes containing Arg at P1. In light of NSP4's low abundance in neutrophils the high kinetic efficiency afforded by PK401 could be of great value by providing sufficient sensitivity for the detection of active enzyme. Future studies will be used to determine whether NSP4 is associated with neutrophil activation and NET formation, similarly to NE, CatG and PR3 [29]. Our procedure confirms the usefulness of HyCoSuL in providing a valuable route to the development of highly selective inhibitors utilizing non-prime pocket recognition elements.

## Supporting Information

S1 Text Contains described in details methods, chemical compound analysis, amino acids structures (Fig A). (PDF)
