# Supplementary material for: Design of a Selective Substrate and Activity Based Probe for Human Neutrophil Serine Protease 4
Source: PLoS One. 2015 Jul 14;10(7):e0132818. doi: 10.1371/journal.pone.0132818 (PMC4501687; doi:10.1371/journal.pone.0132818)
Supplement: S1 Text — (PDF) [file pone.0132818.s001.pdf]

Supporting Material

Full title: Design of a selective substrate and activity based probe for human neutrophil serine protease 4

Short title: Selective NSP4 probe

Paulina Kasperkiewicz[1,2], Marcin Poreba[1], Scott J. Snipas[2], S. Jack Lin[3], Daniel Kirchhofer[3], Guy S. Salvesen[2*] and Marcin Drag[1*]

[1]Division of Bioorganic Chemistry, Faculty of Chemistry, Wroclaw University of Technology, Wyb. Wyspianskiego 27, Wroclaw 50-370, Poland
[2]Program in Cell Death and Survival Networks, Sanford-Burnham Medical Research Institute, 10901 North Torrey Pines Road La Jolla, CA 92024, USA
[3]Dept. of Early Discovery Biochemistry, Genentech Inc., 1 DNA Way, South San Francisco, CA 94080, USA.

*corresponding author:
marcin.drag@pwr.edu.pl (MD)
gsalvesen@sanfordburnham.org (GS)

Materials and Methods

Chemicals were obtained from commercial suppliers and used without further purification. To libraries and individual substrates synthesis, Rink amide RA resin (particle size 200-300 mesh, loading 0.7 mmol/g, Iris Biotech GmbH), Fmoc-protected amino acids (purity > 99% Iris Biotech GmbH, Bachem, Creosalus, Santa Cruz, PepChem), *N*-hydroxybenzotriazole (HOBt purity > 98%, Creosalus), diisopropylcarbodiimide (DICI, peptide grade, Iris Biotech GmbH), *N,N*-diisopropylethylamine (DIPEA, peptide grade, VWR), *O*-benzotriazole-*N,N,N',N'*-tetramethyl-uronium-hexafluoro-phosphate (HBTU, peptide grade, Iris Biotech GmbH), 2-(1-H-7-azabenzotriazol-1-yl)-1,1,3,3-tetramethyl uranium hexafluoro-phosphate methanaminium (HATU, peptide grade, Iris Biotech GmbH), 2,4,6trimethylpyridine (collidine, peptide grade, Sigma Aldrich), *N,N'*-dimethylformamide (DMF, peptide grade, J.T. Baker), dichloromethane (DCM, pure for analysis, POCh), methanol (MeOH, pure for analysis, POCh), acetonitrile (ACN, HPLC gradient grade, Sigma Aldrich), diethyl ether (Et_2O, pure for analysis, POCh), piperidine (PIP, purity > 99%, Iris Biotech), trifluoroacetic acid (TFA, purity 99%, Iris Biotech GmbH), triisopropylsilane (TIPS, purity 99%, Sigma Aldrich), acetic acid (AcOH, purity >98%, POCh), phosphorus pentoxide (P_2O_5, purity 98%, POCh) were used. Individual substrates, inhibitor and ABP were purified by HPLC on a Waters M600 solvent delivery module with a Waters M2489 detector system using a semipreparative column (Discovery BIO Wide Pore C8-10, Supelco Analytical). The solvent composition was as follows: phase A (water/0.1% TFA, v/v) and phase B (ACN/H_2O 80%/20% (v/v) with 0.1% of TFA (v/v)). The purity of each individual compound was confirmed by analytical HPLC using a Supelcosil C8, 5 µm particle size, L × I.D. 25 cm × 2.1 mm column (Supelco Analytical). Finally, the molecular weight of each compound was confirmed by mass spectrometry on High Resolution Mass Spectrometer WATERS LCT Premier XE with Electrospray ionization (ESI) and Time of Flight (TOF). Data files for these studies can be found in the Supplementary Data section. CatG, NE were purchased from Biocentrum, Krakow, Poland and PR3 were purchased from Athens Research Technology, Athens, Georgia, USA. Bolt 4-12% Bis-Tris P lus Gels, 10-well, Bolt MESS DS Running Buffer (20x), Bolt Transfer

Buffer (20x) were purchased from Thermo. Nitrocellulose membrane (0.2μm) was obtained from Bio-Rad.

Library synthesis

Synthesis of P1 Arg (in Arg-HyCoSuL) was carried out in a similar manner as described previously [1-3]. The peptide chain was elongated with standard Fmoc-synthesis procedure. 1eq of Rink Amide resin (13g, 9.1 mmol, 0.7mmol/g, mesh 200-300) was swollen in a glass reaction vessel in DCM, stirred gently once per 10 minutes for an hour. The resin was washed three times with DMF, the Fmoc protecting group was removed with 20% piperidine in DMF with three washes for 5, 5 and 25 minutes, followed by gently washing with DMF (6x). A ninhydrin test was performed to confirm amine group deprotection. In the next step 3eq of Fmoc-ACC-OH were preactivated with 3eq of HOBt and 3eq of DICI in DMF, and after 5 minutes the mixture was poured into the resin. The reaction was carried out by gentle stirring for 24 hours in room temperature. Next the resin was washed three times with DMF and Fmoc-ACC-OH coupling was repeated with 1.5 eq of reagents to increase loading efficiency. After 24 hours, the resin was washed with DMF and coupling was confirmed by a ninhydrin test. The Fmoc group was removed as described above, resin was washed gently and 3 eq of Fmoc-Arg(Pbf)-OH was preactivated with 3 eq of HATU and 3eq of collidine in DMF for 5 minutes, followed by addition to the resin. The reaction was agitated for 24 hours in room temperature and then a second coupling was carried out with 1.5 eq of reagents to increase the loading efficiency.

To cap unreacted aminocoumarin reporter groups, the resin was treated with 10 eq of acetic acid, 10 eq of 3-nitro-1,2,4-triazole and 10 eq of DICI in DMF for 24 hours, followed by addition of 2 eq of DIPEA for 3 hours [3]. Next the amine group from Arg was deprotected with 20% piperidine in DMF and washed with DMF (6x), DCM (3x) and MeOH (3x) and dried over P_2O_5 overnight. Resin was divided in 120 portions and 48 of these portions were added to 48 wells of solid phase peptide synthesis reactor [1, 2, 4]. The resin was swollen with DCM for an hour, shaking once every 10 minutes. In the next step, resin was washed with DMF and 3eq of each of 48 different amino acids (separately in Eppendorf tubes) were preactivated with 3eq of HOBt and 3eq of DICI in a minimal amount of DMF for 3 minutes and added to the glass SPPS reactor wells. The reaction was carried out for 3 hours, followed by

washing with DMF (3x). The Fmoc-protecting group was removed, resin was washed DMF (6x) and 5eq of isokinetic mixture of natural amino acids (omitting Cys and substituting Nle for Met) was preactivated with 5eq of HOBt and 5eq of DICI for 5 minutes and then added to the resin. The Fmoc protecting group was removed with 20% PIP/DMF, resin washed with DMF (6x) and the isokinetic mixture was attached to the P4 position as described above. After Fmoc group removal, the free amine group was protected with 5eq of acetic acid, 5eq of HBTU and 5eq of DIPEA in DMF. After acetylation was complete (1 hour), resin was washed with DMF (3x), DCM (3x) and MeOH (3x) and dried over P_2O_5 overnight.

Finally, tetrapeptide derivatives were cleaved from the resin with a standard procedure, treating the resin with TFA:TIPS:H_2O (95:2.5:2.5, v/v/v) for 2 hours in room temperature, shaking once per 10 minutes. Supernatants were collected in Falcon 15mL conical-bottom centrifuge tubes and the resin was washed once with TFA:TIPS:H_2O (95:2.5:2.5, v/v/v). For each substrate/reaction well both supernatant fractions were combined and substrates were precipitated in cold Et_2O for 30 minutes in -20°C. Then samples were centrifuged (5 minutes, 4°C, 4400 rpm), supernatants were decanted and the precipitates were washed with 10mL of Et_2O, then centrifuged (5 minutes, 4°C, 4400 rpm) and again supernatant was decanted. The crude precipitate was dissolved in ACN:H_2O (40:60) and lyophilized. Compounds as white fluffy powders were dissolved in DMSO to 20mM final concentration, and stored at -80°C until use.

The P2 library with remaining 72 amino acids was synthesized in an analogous manner. For P3 and P4 library synthesis, similar procedure was applied, however in the P3 library isokinetic mixtures were attached in the P2 and P4 positions and in the P4 library these mixtures were coupled in the P2 and P3 positions respectively.

Library screening

NSP4 activity was tested on each sublibrary by measuring the increase of fluorescence signal over time for using a plate reader with 355/460nm (cutoff 455nm) for 1000s in 50 mM Hepes, 0.1 M NaCl, 0.1% TRITON-X100, pH 7.45 buffer.

The final substrate library concentration in each well was 100μM, enzyme concentration for P2 and P4 sublibraries was 326.7nM, and 217.8nM for the P3 sublibrary. Enzyme was preincubated with buffer for 20 minutes at 37°C and added to plate wells containing library components. Relative fluorescence units/second (Rfu/s) measurements were measured using a Spectra Gemini Max plate reader, for a minimum of 1000s at 37°C. The linear portion of the graph was used for further calculations. The Rfu/s values were transformed to percentages by comparison of each substrate hydrolysis velocity with the best amino acid value (100%). NSP4 substrate specificity was presented in histogram format using Excell software.

Individual substrates synthesis

In a glass peptide synthesis vessel, 1eq of Rink Amide resin (0.7 mmol/g, 200-300 mesh) was swollen in DCM for 1 hour stirring once per 10 minutes and then washed with DMF (3x). The Fmoc-protecting group was removed with 20% PIP in DMF for 5, 5 and 25 minutes. Resin was washed with portions of DMF (6x) and deprotection was confirmed by a ninhydrin test (resin beads were navy blue). Afterwards, to the resin 3eq of Fmoc-ACC-OH was loaded with 3eq of HOBt and 3eq of DICI (preactivated for 3 minutes), followed by 24 hours agitation at room temperature. Resin was washed with DMF (3x) and the coupling procedure was repeated with 1.5 eq of Fmoc-ACC-OH, HOBt and DICI. The resin was washed with DMF (3x) and coupling was confirmed by ninhydrin test, followed by Fmoc protecting group removed with 20% PIP/DMF for 5, 5 and 25 minutes. Resin was washed carefully with DMF (6x) and 3eq of first amino acid (Fmoc-Arg(Pbf)-OH) was attached to the resin with 3eq of HATU and 3 eq of collidine as a coupling reagents, stirring gently for 24 hours. The resin was washed with DMF (3x) and loading was repeated with 1.5eq of reagents for additional 24 hours. The resin was washed with DMF (3x) and Fmoc protecting group was removed with 20% PIP/DMF (5, 5 and 25 minutes) and resin was washed with DMF (6x). To confirm deprotection, a ninhydrin test was performed.

The H_2N-R(Pbf)-ACC resin was treated with 3 eq of preactivated P2 Fmoc-amino acid (Fmoc-Oic-OH or Fmoc-Pro-OH) with 3eq of HOBt and 3eq of DICI. Resin was stirred gently for 3 hours and loading was confirmed with a ninhydrin test. The Fmoc protecting group was removed as described above.

Amino acids at P3 (Fmoc-Phe(guan(Boc)$_2$)-OH or Fmoc-Val-OH) and at P4 (Fmoc-Ile-OH or Fmoc-Arg(Pbf)-OH) positions were attached as described for P2 position.

Acetylation of H_2N-P4-P3-P2-Arg-ACC was carried out with 5eq of AcOH, 5eq of HBTU and 5eq of DIPEA, stirred gently for 45 minutes at room temperature. The resin was washed carefully with DMF (4x), DCM (3x) and MeOH (3x), and dried over P_2O_5 overnight.

ACC-labeled peptides were cleaved from the resin using a standard procedure, treating the resin with TFA:TIPS:H_2O (95:2.5:2.5, v/v/v) for 2 hours at room temperature, shaking once every 10 minutes. Supernatants were collected in Falcon 15mL conical-bottom centrifuge tubes and resin was washed with TFA:TIPS:H_2O (95:2.5:2.5, v/v/v). Supernatants were combined and substrates were precipitated in cold Et_2O for 30 minutes at -20°C. The samples were centrifuged, supernatants were decanted and the pellets were washed with 5mL of Et_2O. The centrifugation and decantation procedure was repeated to obtain crude ACC substrates that were dried at room temperature. These substrates were dissolved in 0.7 ml of DMSO and compounds were purified on HPLC (Waters, column: Spherisorb, 5 µm particle size, L × I.D. 25 cm × 4.6 mm) in H_2O:ACN gradient and lyophilized. Compounds were recovered as white fluffy powders were dissolved in DMSO to 20mM final concentration, and stored at -80°C until use.

Activity Based Probe Synthesis

Biotin-labeled peptide sequence synthesis fragment (Biot-Ahx-hCha-Phe(guan)-Oic-OH)

2-chlorotrityl chloride resin (100mg, loading 1.6 mmol/g) dry DCM was added in a glass peptide synthesis vessel and resin was swollen for 1 hour shaking once each 10 minutes. The resin was washed three times with dry DCM, following by addition of 3eq of Fmoc-Oic-OH (0.48 mmol) and 6 eq of DIPEA (0.96 mmol) dissolved in a minimal volume of DCM. Resin was gently stirred under argon atmosphere for 3 hours, then filtered and washed three times with DMF. The Fmoc-protecting group was removed with 20% piperidine in DMF (5, 5 and 25 minutes), and the resin was

washed with DMF (6x). In the next step, 2.5 eq of Fmoc-Phe(guan(Boc)$_2$)-OH (0.4 mmol) was preactivated for three minutes with 2.5 eq of HOBt (0.4 mmol) and 2.5 eq of DICI (0.4 mmol) in DMF and poured into the resin. In the same manner Fmoc-hCha-OH and Fmoc-6-Ahx-OH amino acids were attached to the resin. After Fmoc-protecting group removal (at 6-Ahx-hCha-Phe(guan)-Oic-resin), 5eq of biotin (0.8 mmol), 5eq of HATU (0.8 mmol), and 5eq of DIPEA (0.8 mmol) were preactivated (5 minutes) in DMSO:DMF (1:1, v/v) solution and added to the resin. The coupling rection was carried out for 2 hours in room temperature. The resin was washed with DMSO (3x), DMF (3x), DCM (3x) and MeOH (3x) and dried over P_2O_5. Product was cleaved from the resin with solution of TFE:AcOH:DCM (1:1:4; v/v/v), for 1 hour, stirred at room temperature. The filtrate was collected, the resin was washed with cleaving mixture and filtrate was combined with previous portion and concentrated on a rotary evaporator under reduced pressure. Then crude product was dissolved in ACN:H_2O (1:1, v/v) and lyophilized. Crude product was recovered as a white powder and used in further synthesis without purification.

Biot-Ahx-hCha-Phe(guan)-Oic-Arg^P(OPh)$_2$ synthesis (PK401)

To a round bottom flask fitted with a stirring bar, 1eq of Biot-Ahx-hCha-Phe(guan(Boc)$_2$)-Oic-OH, 1 eq of H_2N-R(Boc$_2$)^P(OPh)$_2$, 1 eq of HATU, and 3 eq of collidine (pH was adjusted to 7-8) in DMF were added and stirred for 2 hours at room temperature. The reaction was monitored by analytical HPLC. Crude compound was purified by HPLC (Discovery BIO Wide Pore C8-10, semi-preparative column) and lyophilized. Purity of the compound was confirmed by analytical HPLC (Discovery BIO Wide Pore C8, analytical column). Finally, Boc protecting groups were removed with TFA:DCM (1:1, v/v) within 30 minutes - the reaction progress was monitored once by analytical HPLC. Crude compound was then purified by HPLC (Discovery BIO Wide Pore C8-10, semi-preparative column) and lyophilized. Purity of the compound was confirmed by analytical HPLC and HRMS.

SDS-page and Western Blot

NSP4visualization by PK401

A standard SDS broad spectrum marker (Biorad) was used (Fig. 4A, lane 1). Two Eppendorf tubes with 100nM of NSP4 were incubated with Ac-IVPR-CMK for 30 minutes at 37°C and then treated with PK401 at 2000nM and 1000nM for an additional 20 minutes at 37°C (Fig. 4, lanes 2 and 3). Separately, 100nM enzyme was incubated with 2000nM, 1000nM, 100nM, 10nM, and 1 nM of PK401 for 20 minutes in 37°C (Fig. 4, lanes 4, 5, 6, 7, 8). As a control 100nM PK401 alone was incubated with assay buffer (Fig. 4, lane 9). Samples were heated for 5 minutes at 95°C in 5xSDS buffer and 20µL of each was run at the Bolt 4-12% Bis-Tris Plus Gels, 10-well (165V, 36min), followed by transfer to nitrocellulose membrane (10V, 60min) in MES running buffer. The membrane was blocked with 1.5% BSA in TBST overnight at 4°C and treated with IRDye800CW Streptavidin (dilution 1:10000 with 1,5% BSA in TBST) for 45 minutes. Then enzyme was visualized using a Li-cor fluorescence imager and Odyssey Molecular Modeling Software.

NSP's visualization by PK401

A standard SDS broad spectrum marker (Biorad) was used. 100nM of each NE< PR3, CatG and NSP4 were treated with PK401 at 100nM for 10 minutes at 37°C. Separately, 100nM of NE, PR3, CatG and NSP4 were incubated with buffer for 10 minutes in 37°C. Samples were heated for 5 minutes at 95°C in 4xSDS buffer and 21µL of each was run at the Bolt 4-12% Bis-Tris Plus Gels, 10-well (165V, 36min), followed by transfer to nitrocellulose membrane (10V, 60min) in MES running buffer. The membrane was blocked with 1.5% BSA in TBST overnight at 4°C and treated with IRDye800CW Streptavidin (dilution 1:10000 with 1,5% BSA in TBST) for 45 minutes. Then PK401 was visualized using a Li-cor fluorescence imager and Odyssey Molecular Modeling Software.

Analysis data of synthesized substrates and PK401

PK431, Ac-hCha-Phe(guan)-Pro-Arg-ACC

27.0% yield. HRMS (m/z): [M^+] calcd for C44H60N12O8, 884.4657; found, 885.4753

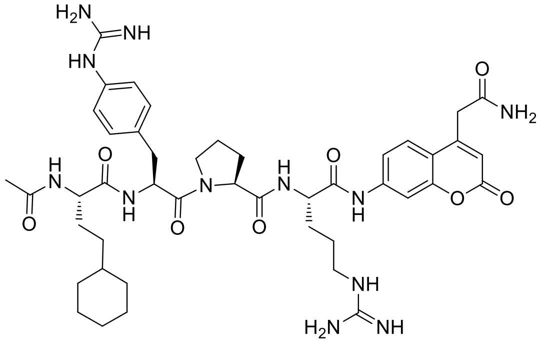

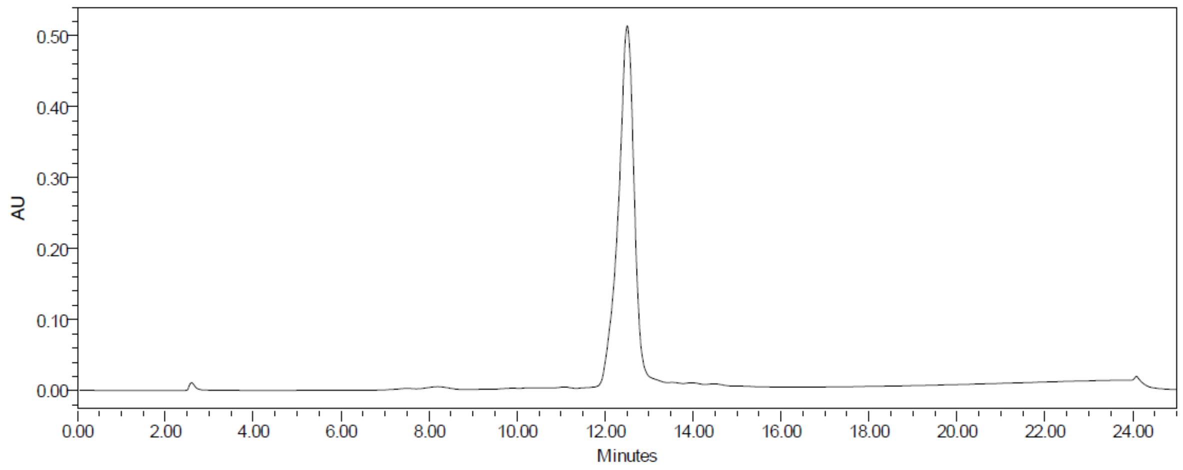

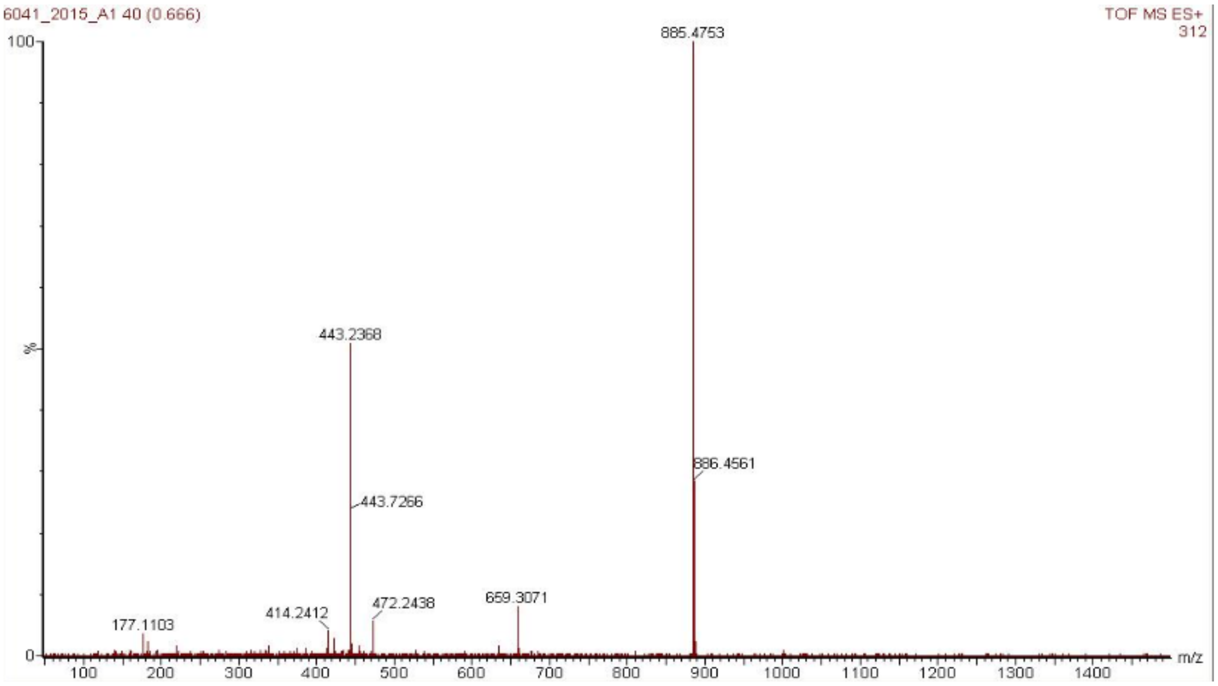

PK421, Ac-hCha-Phe(guan)-Oic-Arg-ACC

25.0% yield. HRMS (m/z): [M^+] calcd for C48H66N12O8, 938.5127; found, 939.6243

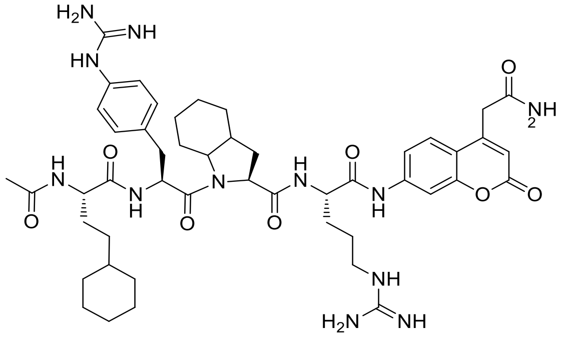

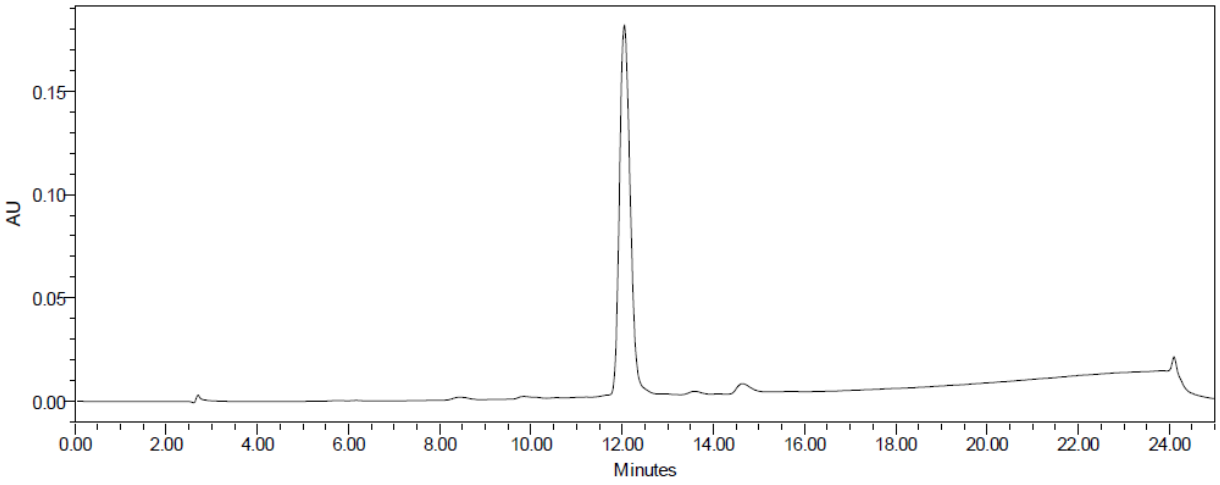

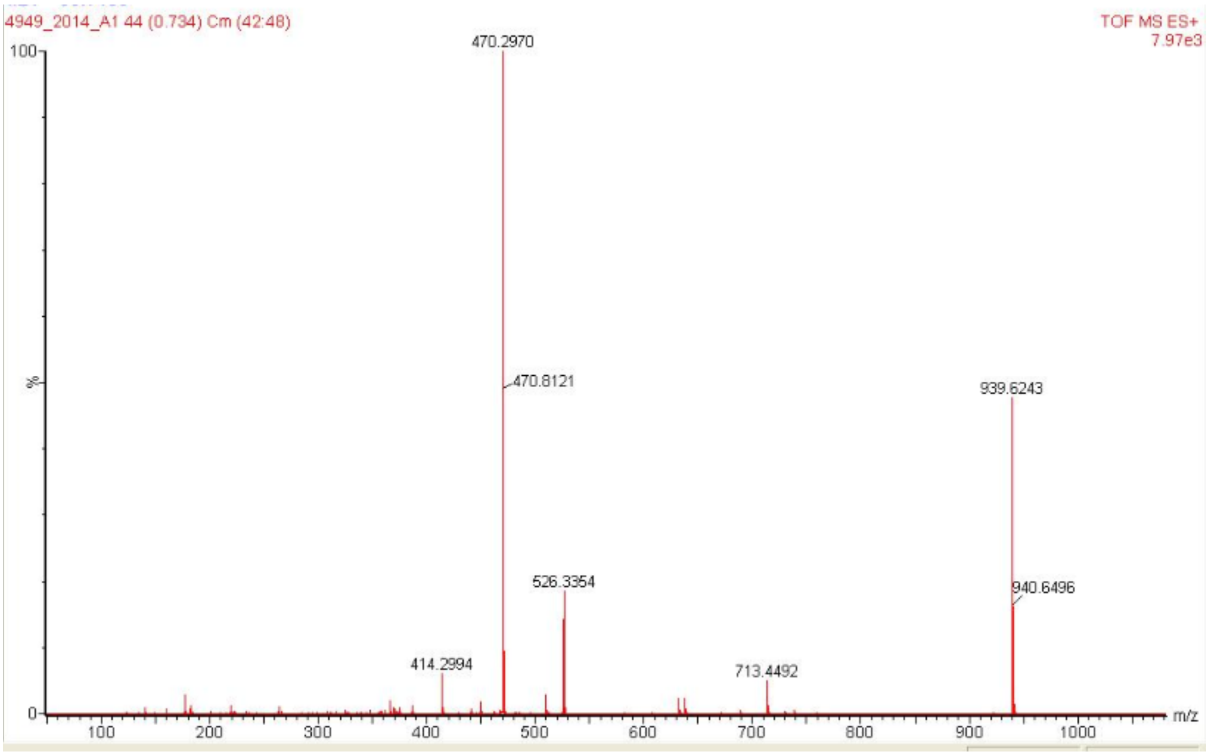

PK417, Ac-Ile-Val-Pro-Arg-ACC

39.0% yield. HRMS (m/z): [M^+] calcd for C35H51N9O8, 725.3861; found, 726.3926

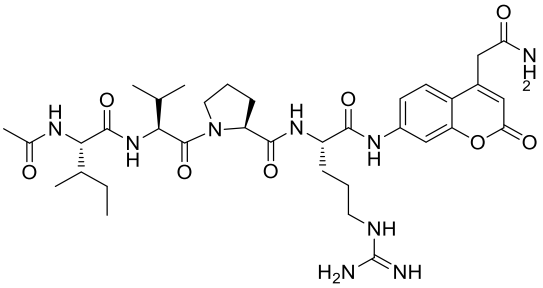

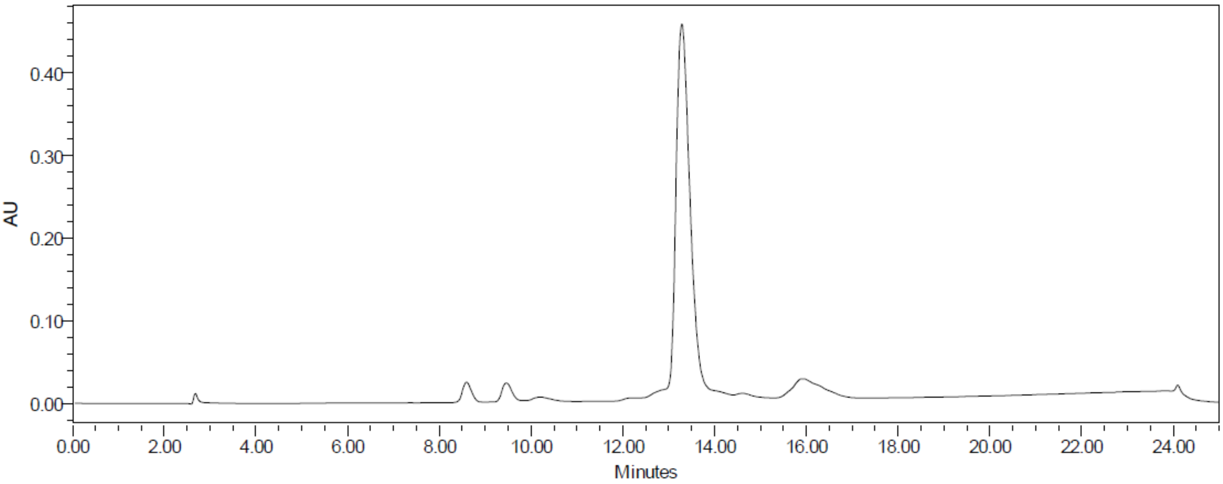

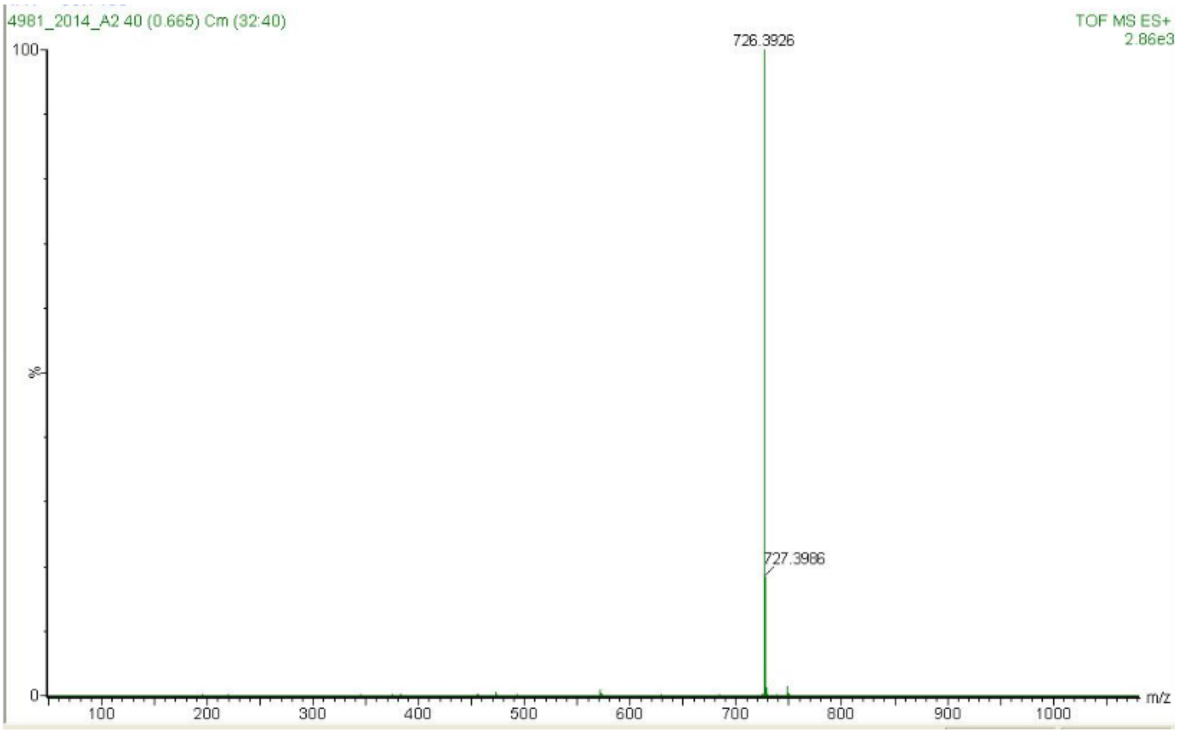

PK418, Ac-Arg-Val-Pro-Arg-ACC

30.0% yield. HRMS (m/z): [M+] calc for C35H52N12O8, 768,4031, found, 769.4095

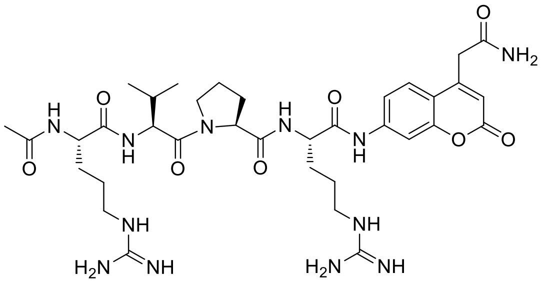

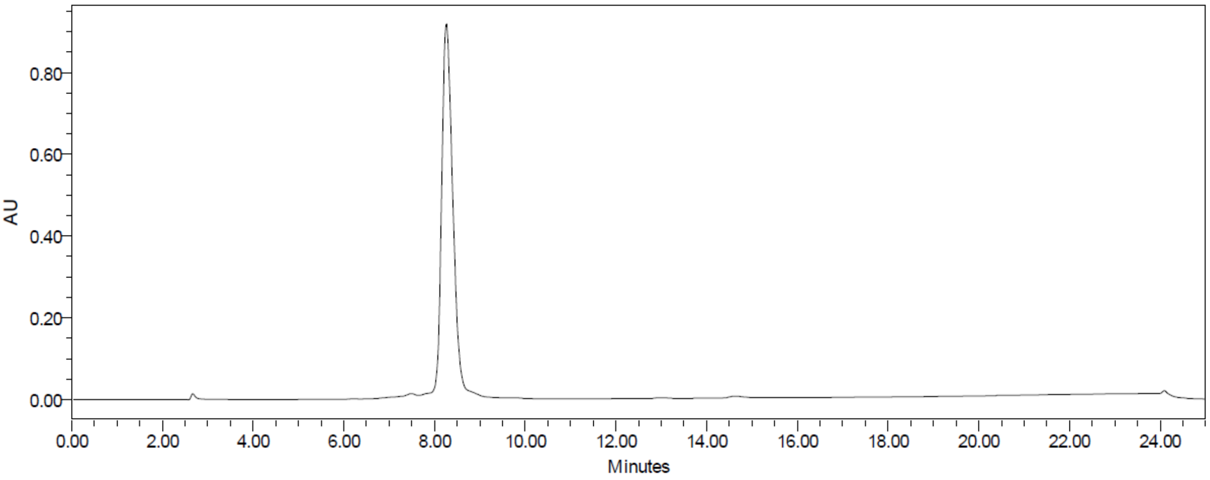

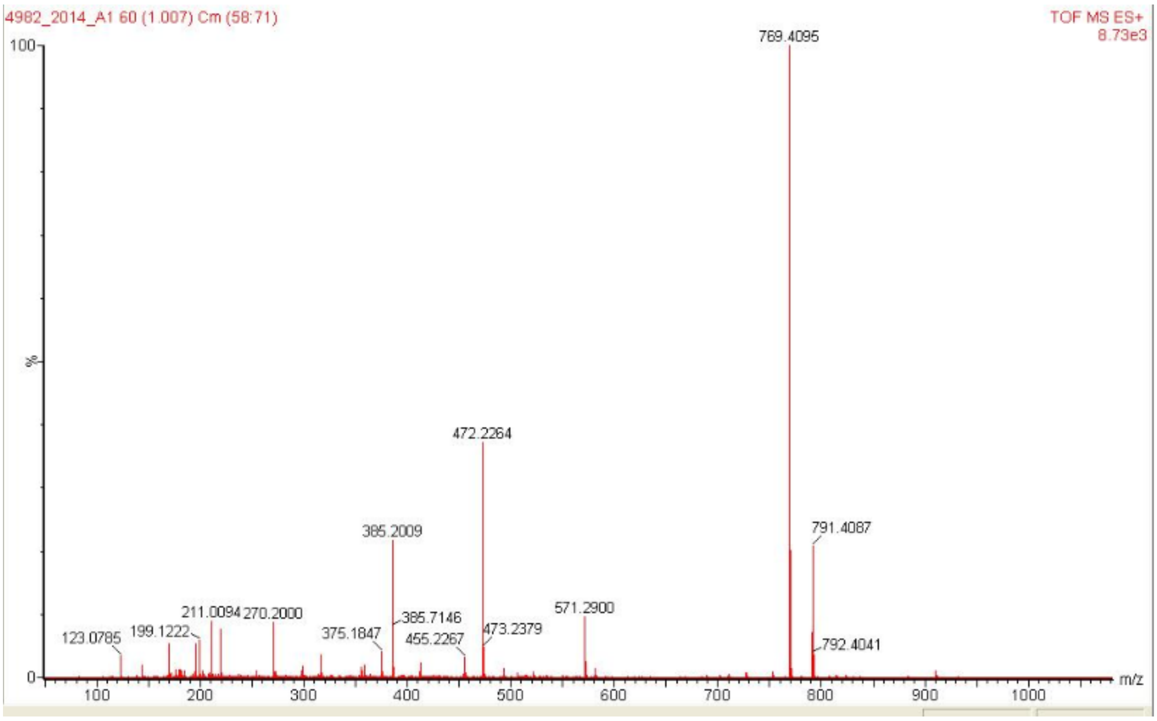

PK401, Biot-Ahx-hCha-Phe(guan)-Oic-Arg-ACC

30.0% yield. HRMS (m/z): [M+] calc for C62H90N13O9PS, 1223.6443, found, 1224.6530

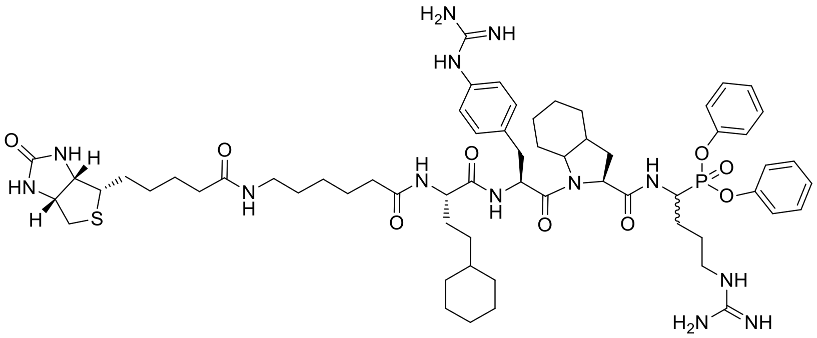

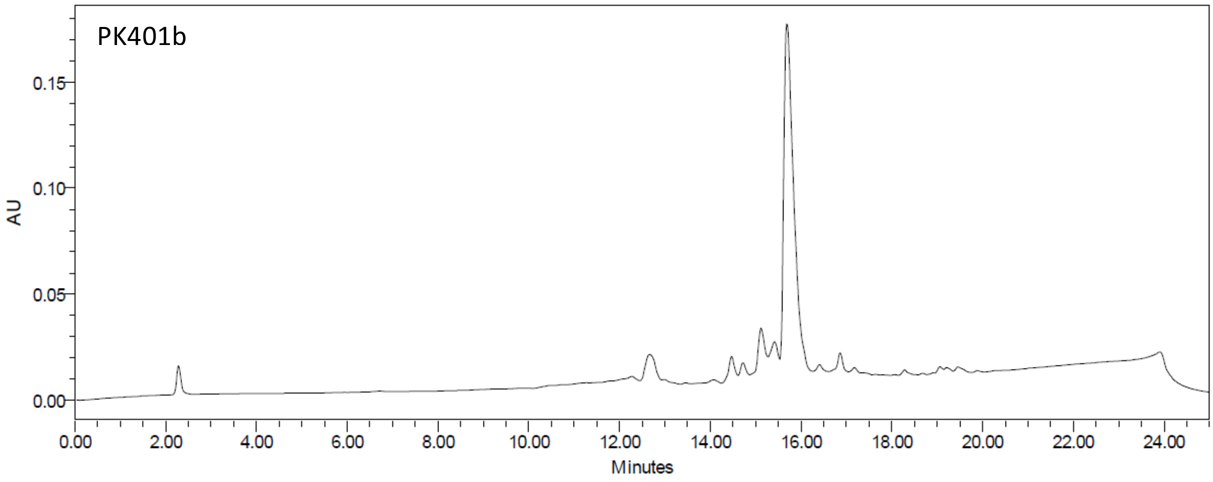

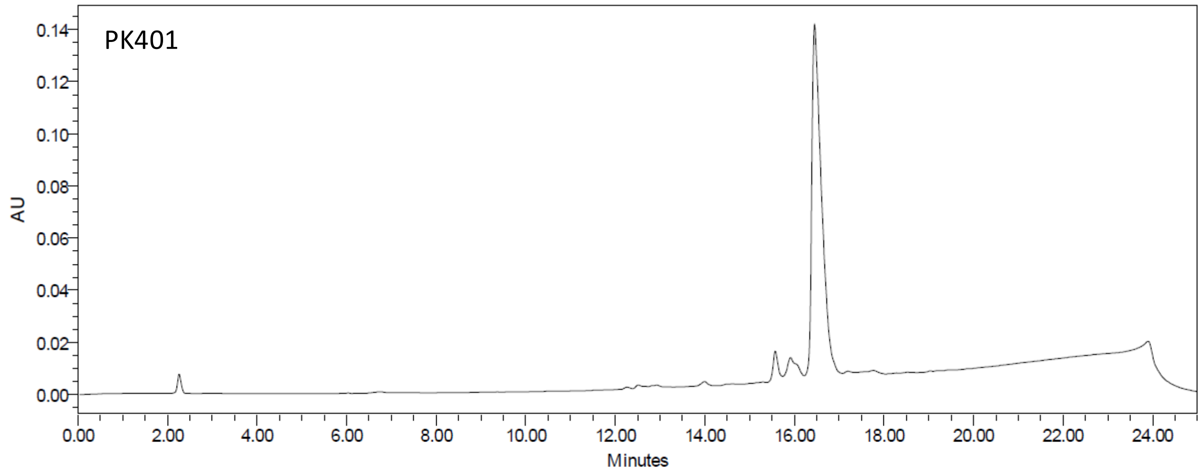

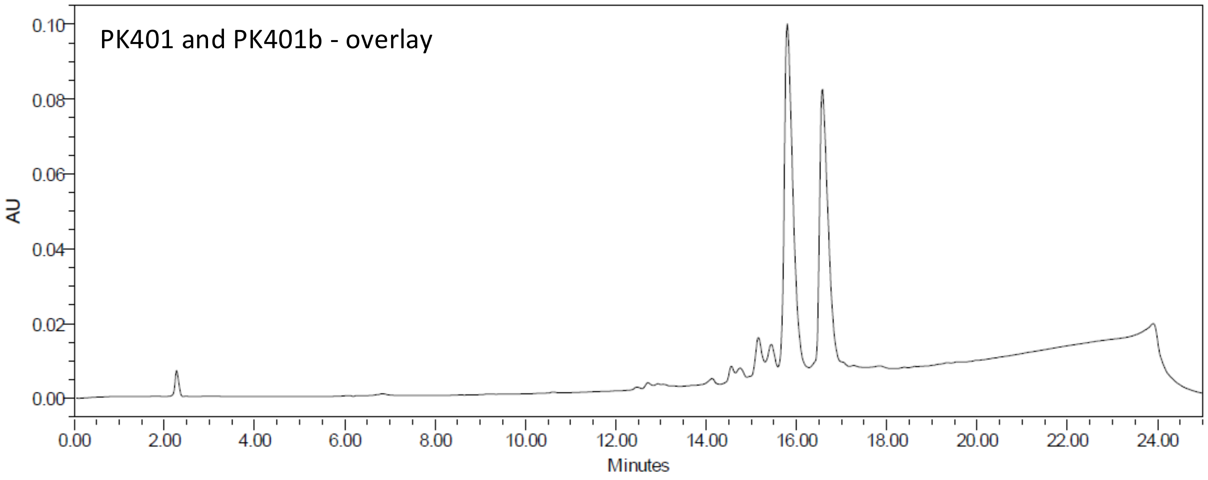
PK401 and PK401b - overlay
AU
Minutes
0.10
0.08
0.06
0.04
0.02
0.00
0.00
2.00
4.00
6.00
8.00
10.00
12.00
14.00
16.00
18.00
20.00
22.00
24.00

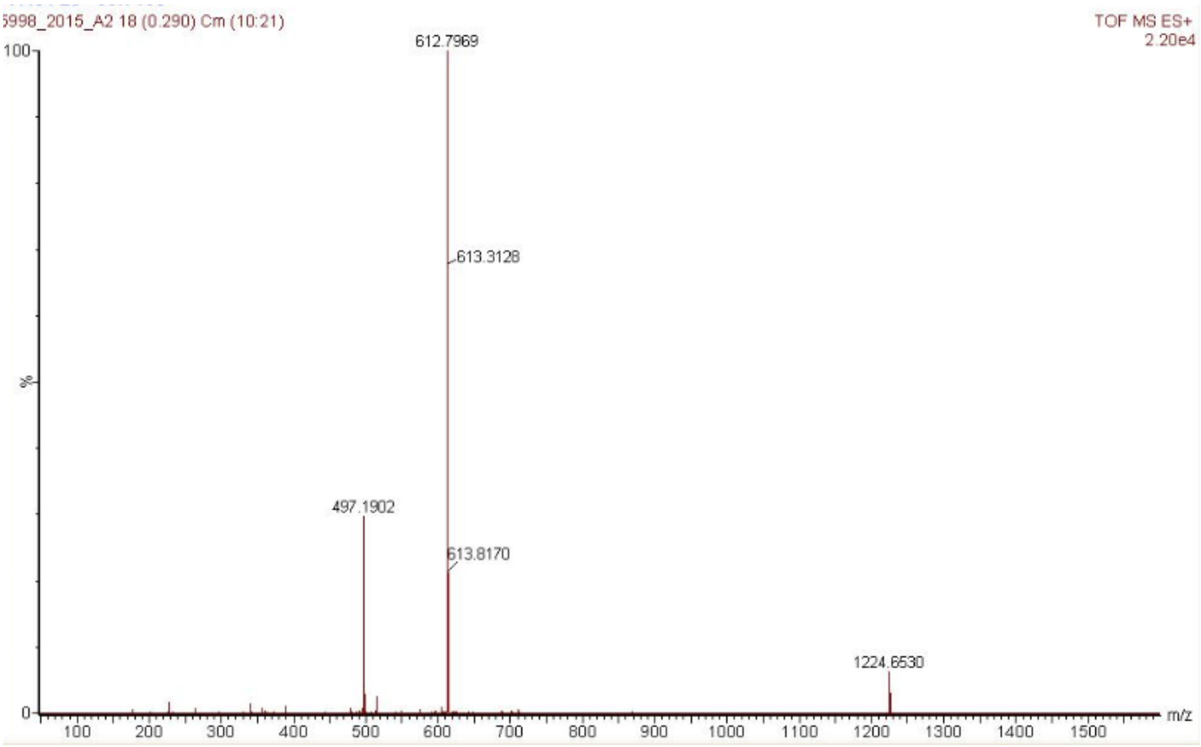
5998_2015_A2 18 (0.290) Cm (10:21)
TOF MS ES+
2.20e4
612.7969
613.3128
497.1902
613.8170
1224.6530
100
%
0
m/z
100
200
300
400
500
600
700
800
900
1000
1100
1200
1300
1400
1500

Fig. A. Structures of used natural and unnatural amino acids

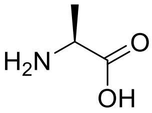
L-Ala

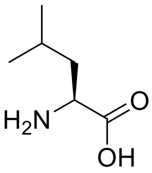
L-Leu

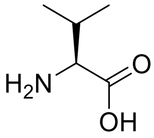
L-Val

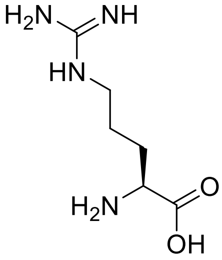
L-Arg

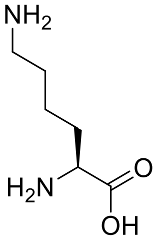
L-Lys

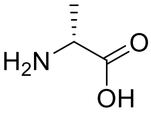
D-Ala

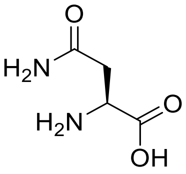
L-Asn

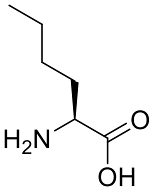
L-Nle

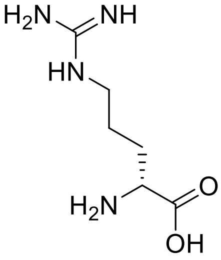
D-Arg

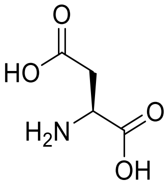
L-Asp

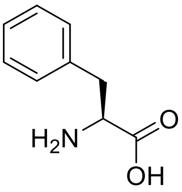
L-Phe

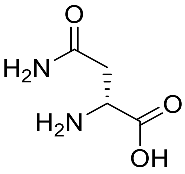
D-Asn

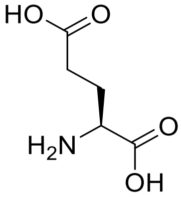
L-Glu

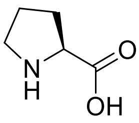
L-Pro

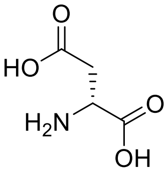
D-Asp

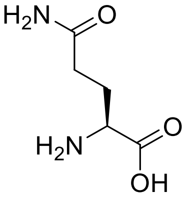
L-Gln

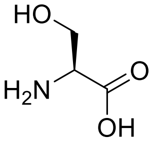
L-Ser

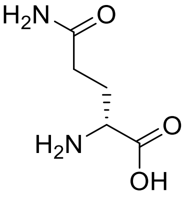
D-Gln

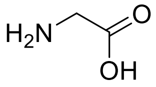
L-Gly

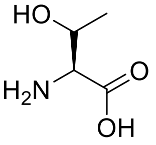
L-Thr

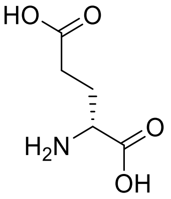
D-Glu

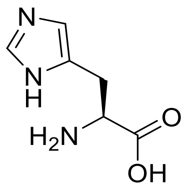
L-His

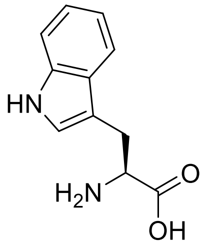
L-Trp

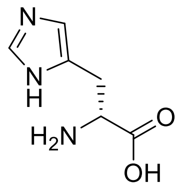
D-His

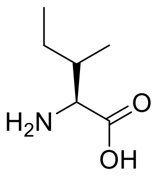
L-Ile

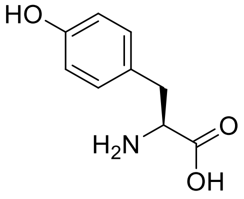
L-Tyr

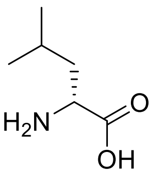
D-Leu

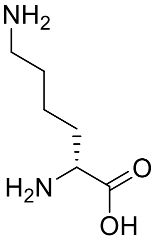
D-Lys

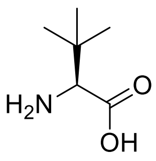
L-Tle

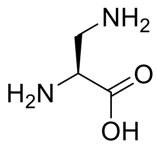
L-Dap

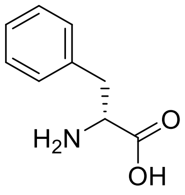
D-Phe

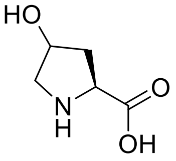
L-hPro

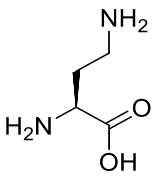
L-Dab

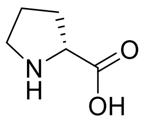
D-Pro

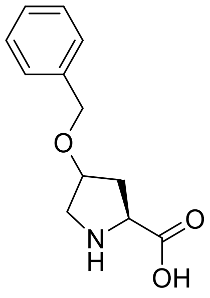
L-Hyp(O-Bzl)

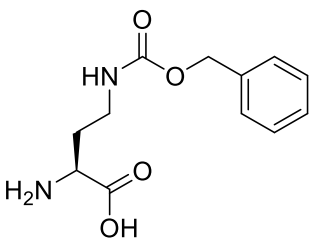
L-Dab(Z)

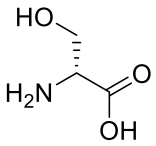

D-Ser

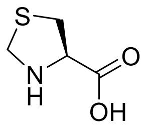

L-Thz

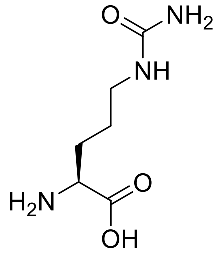

L-Cit

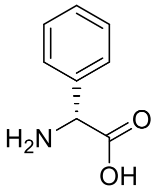

D-Phg

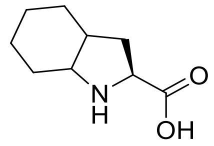

L-Oic

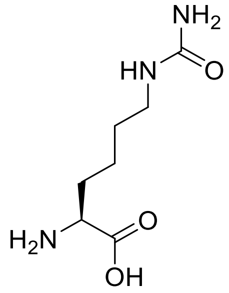

L-hCit

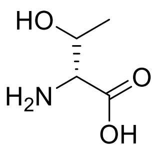

D-Thr

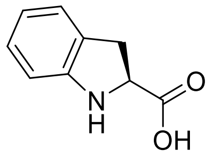

L-Idc

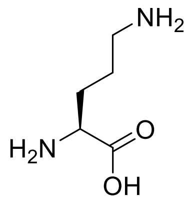

L-Orn

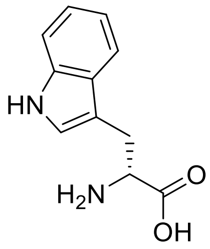

D-Trp

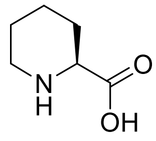

L-Pip

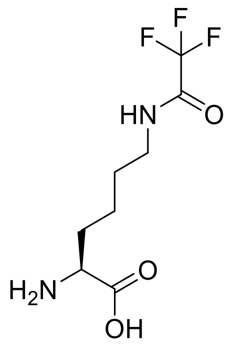

L-Lys(TFA)

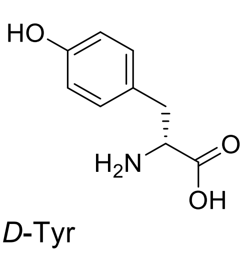

D-Tyr

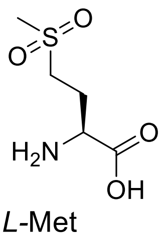

L-Met

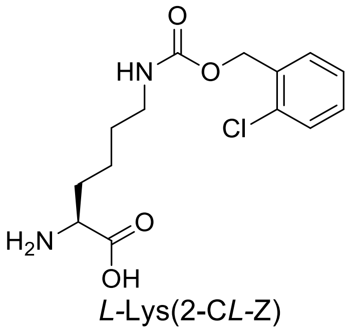

L-Lys(2-C*L*-Z)

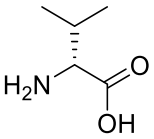

D-Val

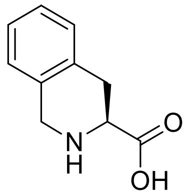

L-Tic

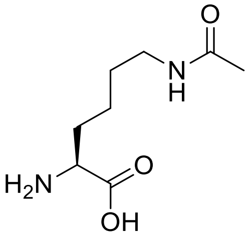

L-Lys(Ac)

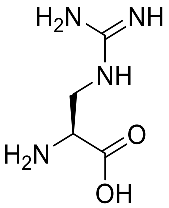

L-Agp

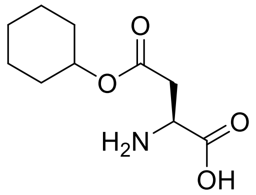

L-Asp(O-cHx)

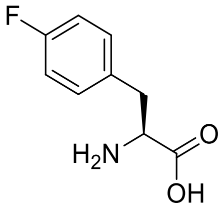

L-Phe(4-F)

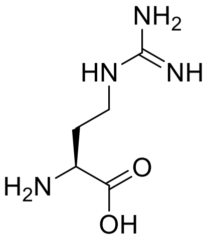

L-Agb

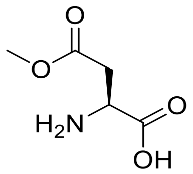

L-Asp(O-Me)

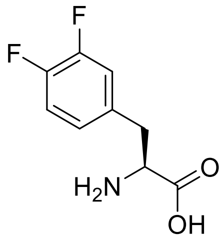

L-Phe(3,4-F_2)

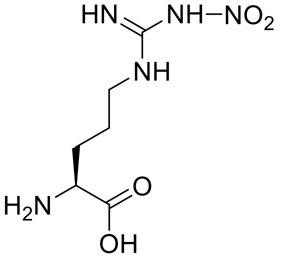

L-Arg(NO_2)

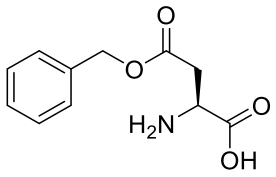

L-Asp(O-Bzl)

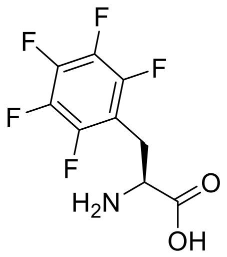

L-Phe(F_5)

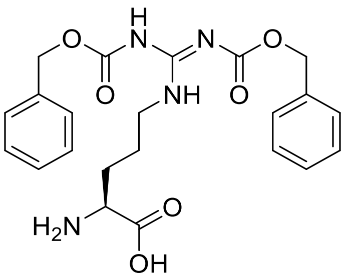

L-Arg$(Z)_2$

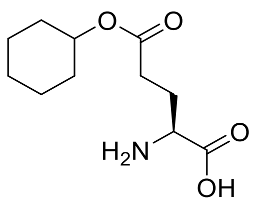

L-Glu(O-cHx)

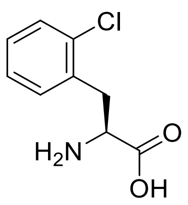

L-Phe(2-Cl)

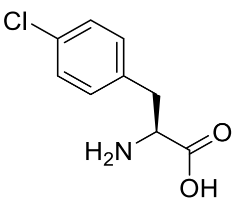

L-Phe(4-Cl)

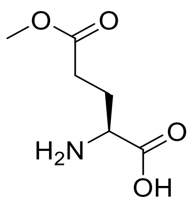

L-Glu(O-Me)

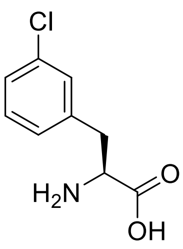

L-Phe(3-Cl)

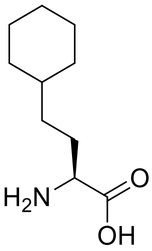

L-hCha

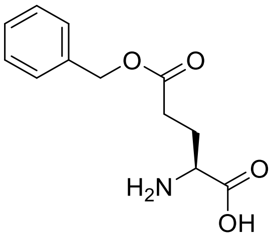

L-Glu(O-Bzl)

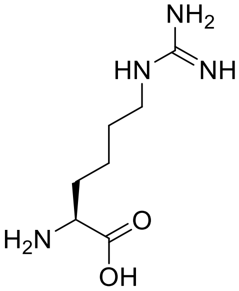

L-hArg

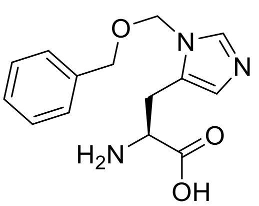

L-His(3-Bom)

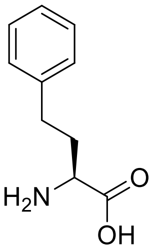

L-hPhe

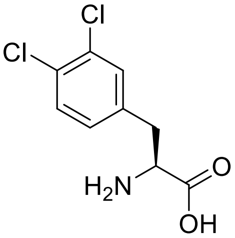

L-Phe(3,4-Cl_2)

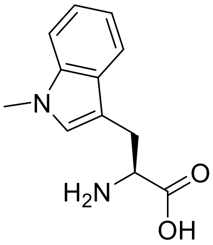

L-Trp(Me)

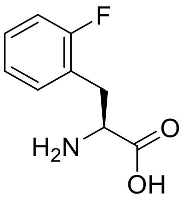

L-Phe(2-F)

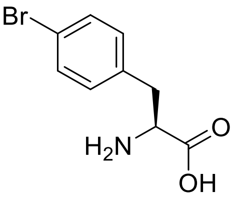

L-Phe(4-Br)

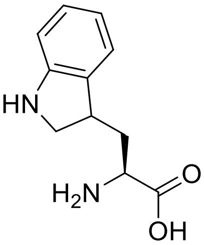

L-Dht

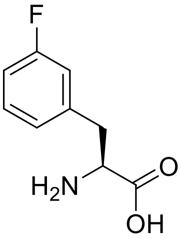

L-Phe(3-F)

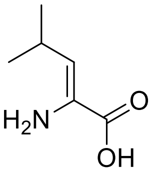

dhLeu

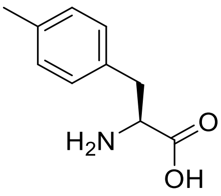

L-Phe(4-Me)

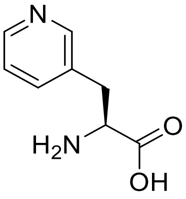

L-3-Pal

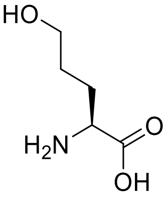

L-Hnv

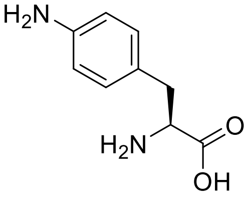

L-Phe(4-NH_2)

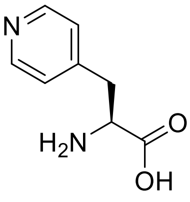

L-4-Pal

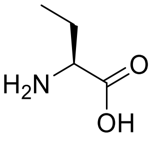

L-Abu

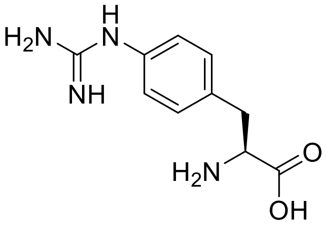

L-Phe(4-guan)

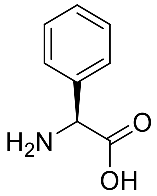

L-Phg

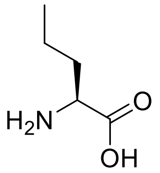

L-Nva

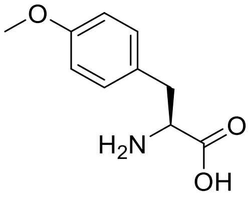

L-Tyr(Me)

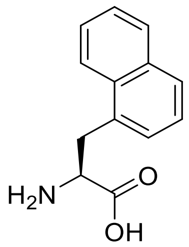

L-1-Nal

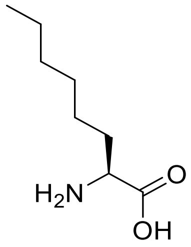

L-2-Aoc

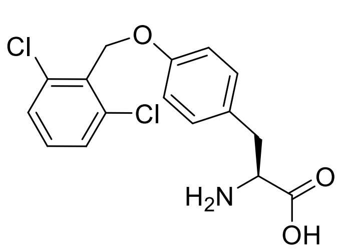

L-Tyr(2,6-Cl_2-O-Bzl)

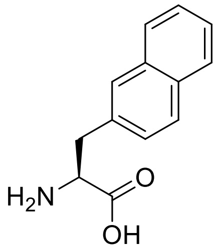

L-2-Nal

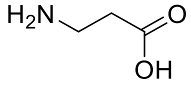

L-β-Ala

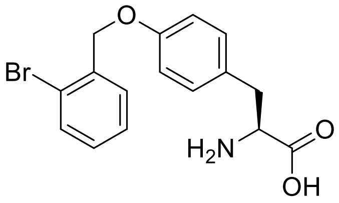

L-Tyr(2-Br-O-Bzl)

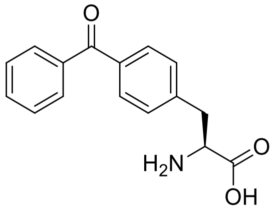

L-Bpa

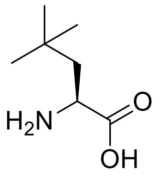

L-NptGly

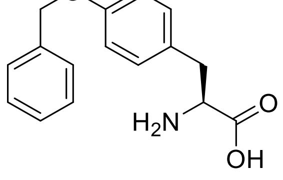

L-Tyr(O-Bzl)

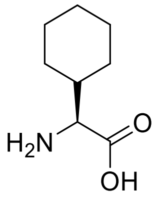

L-Chg

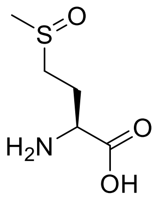

L-Met(O)

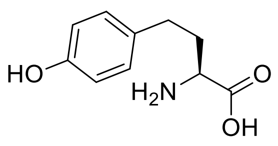

L-hTyr

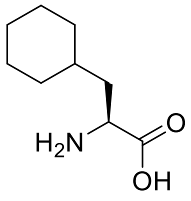

L-Cha

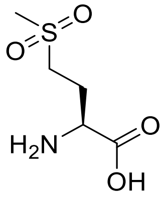

L-Met$(O)_2$

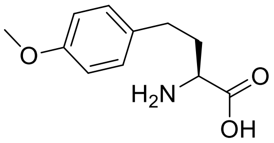

L-hTyr(O-Me)

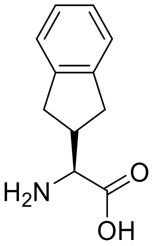

L-Igl

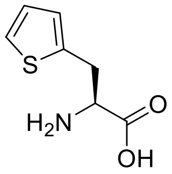

L-Ala(2-Thienyl)

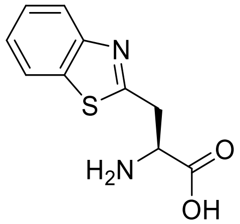

L-Ala(Bth)

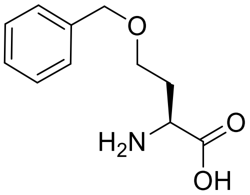

L-Hse(O-Bzl)

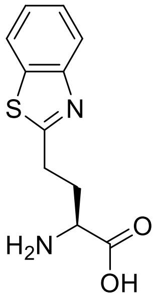

L-Abu(Bth)

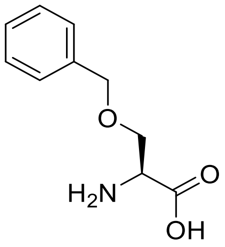

L-Ser(O-Bzl)

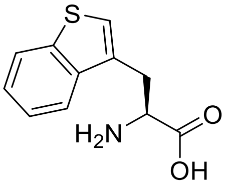

L-Bta

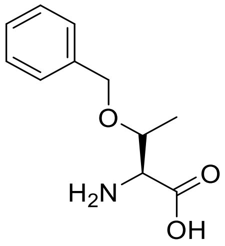

L-Thr(O-Bzl)

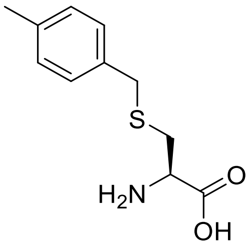

L-Cys(4-MeBzl)

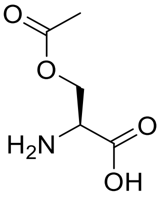

L-Ser(Ac)

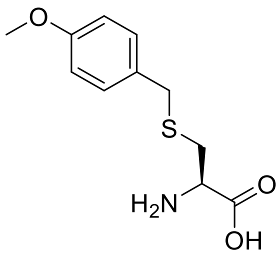

L-Cys(4-Me-O-Bzl)

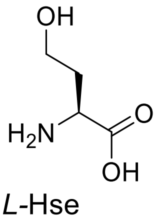

L-Hse

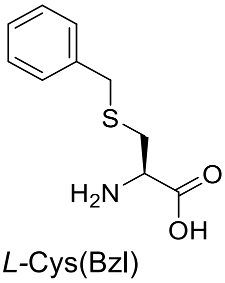

L-Cys(Bzl)

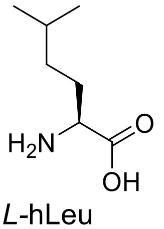

L-hLeu

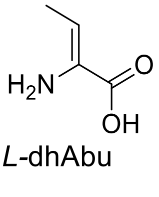

L-dhAbu

References:

1. Kasperkiewicz P, Poreba M, Snipas SJ, Parker H, Winterbourn CC, Salvesen GS, et al. Design of ultrasensitive probes for human neutrophil elastase through hybrid combinatorial substrate library profiling. Proc Natl Acad Sci U S A. 2014;111(7):2518-23.
2. Poreba M, Kasperkiewicz P, Snipas SJ, Fasci D, Salvesen GS, Drag M. Unnatural amino acids increase sensitivity and provide for the design of highly selective caspase substrates. Cell Death Differ. 2014;21(9):1482-92.
3. Maly DJ, Leonetti F, Backes BJ, Dauber DS, Harris JL, Craik CS, et al. Expedient solid-phase synthesis of fluorogenic protease substrates using the 7-amino-4-carbamoylmethylcoumarin (ACC) fluorophore. J Org Chem. 2002;67(3):910-5.
4. Poreba M, Szalek A, Kasperkiewicz P, Drag M. Positional scanning substrate combinatorial library (PS-SCL) approach to define caspase substrate specificity. Methods Mol Biol. 2014;1133:41-59.
